# Translation, Cultural Adaptation, and Validation of the Japanese eHealth Literacy Questionnaire Among Users in a Super-Aged Society: Mixed Methods Study

**DOI:** 10.2196/68529

**Published:** 2025-11-26

**Authors:** Yuh Morimoto, Naotake Yanagisawa, Ryuichi Sawa, Marcellus Nealy, Miwa Sekine, Megumi Ikeda, Kei Matsuno, Tetsuya Takahashi, Katsumi Miyauchi, Hiroyuki Daida

**Affiliations:** 1 Department of Radiological Technology, Faculty of Health Science, Juntendo University, 2-1-1 Hongo Bunkyo-ku, Tokyo, 113-8421, Japan, 81 3813 3111; 2 Medical Technology Innovation Center, Juntendo University, Tokyo, Japan; 3 Department of Physical Therapy, Faculty of Health Science, Juntendo University, Tokyo, Japan; 4 Division of General Education, Faculty of Medicine, Juntendo University, Tokyo, Japan; 5 Department of Medical Education, Faculty of Medicine, Juntendo University, Tokyo, Japan; 6 Faculty of Health Care and Nursing, Juntendo University, Tokyo, Japan; 7 Department of Respiratory Medicine, Juntendo Tokyo Koto Geriatric Medical Center, Tokyo, Japan; 8 Department of Cardiology, Juntendo Tokyo Koto Geriatric Medical Center, Tokyo, Japan

**Keywords:** aging society, digital health literacy, eHealth Literacy Questionnaire (eHLQ), psychometrics, validation study

## Abstract

**Background:**

Japan is likely the world’s leading aging society, and when leveraged effectively, digital health services hold great potential for addressing health care system challenges. Appropriate development and facilitation of these services require the evaluation of users’ digital health literacy. However, the status of digital health literacy in Japan, especially among older adults, remains unclear, which limits efforts to effectively promote such services.

**Objective:**

This study aimed to translate the eHealth Literacy Questionnaire (eHLQ) into Japanese and test the translation’s reliability. Additionally, it aimed to examine eHLQ scores among Japan’s population, compare them across various backgrounds, and explore considerations for applying digital health systems to support Japan’s aging society.

**Methods:**

A sequential exploratory mixed methods design was employed. The translation process prioritized achieving conceptual equivalence with the source language. To evaluate the translation’s reliability, psychometric assessments by survey were conducted based on classical test theory and item response theory. Comparative statistical analysis was used to examine the impact of demographic factors.

**Results:**

A total of 504 individuals aged 18-88 years completed the survey, with 444 responding online and 60 in face-to-face interviews. The Japanese eHLQ showed good internal consistency, with Cronbach *α* values ≥0.78. The comparative fit index was >0.95, standardized root mean residual was ≤0.04, and standardized expected parameter change was ≤0.23, indicating good to acceptable fit. Item response theory analysis found adequate item location, discrimination, and factor loading. Boundary characteristic curve plotting demonstrated that all items fit well. Participants with more frequent digital health service use and better self-reported health status showed higher eHLQ scores across all 7 scales: (1) Using technology to process health information, (2) Understanding of health concepts and language, (3) Ability to actively engage with digital services, (4) Feel safe and in control, (5) Motivated to engage with digital services, (6) Access to digital services that work, and (7) Digital services that suit individual needs.

**Conclusions:**

The Japanese version of the eHLQ is robust and is likely to be a reliable and effective tool for assessing digital health literacy among Japan’s population. There were no notable differences between scores of those aged above and below 65 years, or those with and without chronic disease(s). The findings also suggest that current systems may be tailored to frequent digital health users. The Japanese version of the eHLQ is expected to be used to effectively monitor digital health literacy in Japan’s aging society.

## Introduction

### Background

Rapid advancement of digital technology in medicine has led to the deployment of numerous digital health tools and solutions, transforming health care delivery. Digital health offers various benefits, including improved access to health care, tailored treatment, and reduced costs [1,2]. However, people with limited digital health literacy may struggle to utilize these services, creating disparities in the digital era [3]. Addressing such utilization challenges requires careful development and introduction of technology [4]. Accordingly, health care professionals need robust methods to assess patients’ knowledge, skills, and attitudes regarding digital health technology [4]. Well-tested, multifaceted tools are essential to measure digital health literacy, determine user-specific barriers, and identify methods and resources to facilitate technology use.

The concept of digital health literacy, originally proposed by Norman and Skinner in 2006 as “eHealth literacy,” was defined as “the ability to seek, find, understand, and appraise health information from electronic sources and apply the knowledge gained to addressing or solving a health problem” [5]. Since then, the notion has been refined and expanded [3,6-9]. Norman and Skinner [10] developed the eHealth Literacy Scale (eHEALS), which was the first digital health literacy assessment tool. Available in over 20 languages, eHEALS is the most frequently investigated instrument [11,12]. However, because eHEALS predates smartphones and the widespread use of social networking services, it has limited scope regarding Web 2.0 applications [13,14]. To bridge this gap, additional digital health literacy assessment tools have been developed and are now available [13-20].

### Super-Aged Japanese Society: Health Care Issues

Population aging is a global phenomenon, with “super-aged society” referring to a society with more than 21% of its population being older adults [21,22]. In Japan, currently about one-third of the population is ≥65 years old. This trend leads to increasing numbers of patients with both physical and cognitive challenges [23]. While the average life expectancy is 83.2 years, healthy life expectancy falls short at 73.9 years [24]. Consequently, older adults in Japan typically rely on health care services during the last decade of their lives. The challenges posed by a shortage of health care resources and the burden on Japan’s universal health care system are evident and require urgent solutions [25].

### Background and Rationale for Developing a Japanese Version of the eHealth Literacy Questionnaire

Given the challenges of an aging society, implementing digital health services may alleviate some strain on the Japanese health care system. For example, the increasing number of chronic diseases is one of the major challenges in aging societies, and using digital health services is recommended to properly self-manage these conditions [26-29]. If digital health services are designed to accommodate users’ digital health literacy, more people can benefit, and with targeted support for those facing difficulties, a broader population can be included. However, the status of digital health literacy in Japan, particularly among older adults, remains unclear. To address this, we translated the English version of the eHealth Literacy Questionnaire (eHLQ) into Japanese and applied it to people in Japan. We then evaluated the data to ensure its reliability. The eHLQ was chosen because it is a multifaceted instrument available in over 20 languages with widespread use in North America, Europe, and Asia-Pacific region [18,30-34]. An instrument that has been used around the world is useful because digital health services are often developed by global companies and used worldwide [35]. The eHLQ measures not only the ability to use digital technology but also user experience and users’ perceptions of the services [18]. The multifaceted nature of the eHLQ is useful for evaluating digital health literacy across diverse populations, including those with limited access to digital technology who may recognize such services only indirectly through relatives or the media. In addition, the eHLQ is now used in the international initiative “Health literacy development for the prevention and control of noncommunicable diseases (NCDs)” promoted by the World Health Organization (WHO) [36], with NCDs being common reasons for health care visits among elderly individuals.

### Objectives and Study Scope

This study detailed the translation process to obtain the Japanese version of the eHLQ and presented a rigorous psychometric analysis based on classical test theory and item response theory (IRT). Additionally, it examined digital health literacy within Japan’s population using comparative analysis based on demographic factors. Although the study did not evaluate implementation, we discussed considerations, based on the findings, for the future development and facilitation of digital health services. This study benefits not only health care workers but also developers and providers of digital health systems. In turn, it is also beneficial for general users and their respective communities.

### eHealth Literacy Framework: Conceptual Framework of the eHLQ

**Figure 1.**
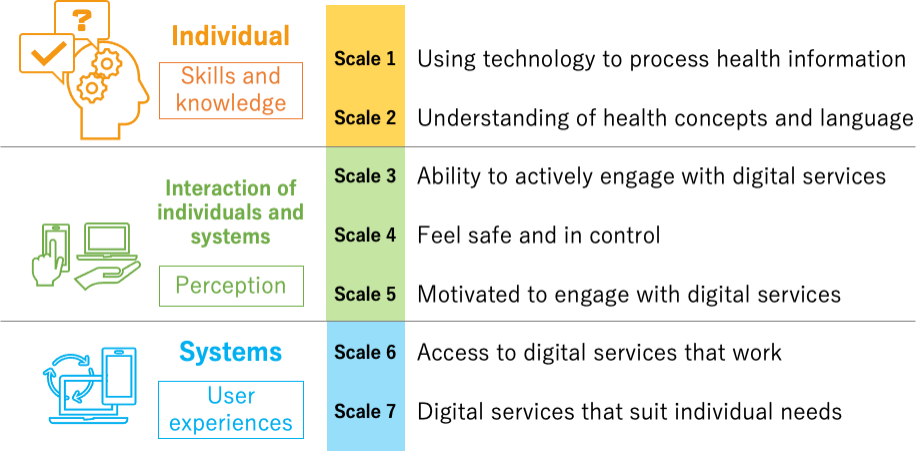
7 Scales of eHealth Literacy Questionnaire (eHLQ).

This study was based on the conceptual framework of “eHLF” (eHealth Literacy Framework) developed by Norgaard et al [37]. The eHLF was conceptualized from real-world observations through multiple workshops [37], which stands out from various other frameworks in the same field [5-9,38]. The eHLQ includes 35 items across 7 scales of the eHLF framework [18]: (1) Using technology to process health information, (2) Understanding of health concepts and language, (3) Ability to actively engage with digital services, (4) Feel safe and in control, (5) Motivated to engage with digital services, (6) Access to digital services that work, and (7) Digital services that suit individual needs. Each item has 4 possible responses: strongly disagree, disagree, agree, and strongly agree, scored from 1 to 4, respectively. Scales 1 and 2 demonstrate skills and knowledge of individuals, scales 3-5 indicate interactions of individuals and systems that influence perceptions, and scales 6 and 7 denote systems that shape user experiences [18,37] (Figure 1).

### Definition of “Digital Health”

While Norman and Skinner first used the term “eHealth” to describe their new concept of literacy, the term “digital health” has been used widely in recent years, including by the WHO and European Commission [39,40]. The term “digital health” encompasses not only eHealth (the use of information and communication technology for health) but also other health-related technologies, serving as an umbrella term [39]. Accordingly, the WHO defines digital health literacy as “*the ability to search, find, understand and evaluate health information from electronic resources and to use the knowledge gained to solve health-related problems*” [41]. In this study, we use the term “digital health” unless “eHealth” is specifically required.

## Methods

### Study Design

This study employed a sequential exploratory mixed methods design [42], containing a qualitative Phase 1 and a quantitative Phase 2a, and was extended with a quantitative Phase 2b. In Phase 1, the English version of the eHLQ was translated into Japanese and culturally adapted. Phase 2a involved its psychometric assessment using classical test theory and IRT approaches. In Phase 2b, snapshots of digital health literacy in Japan were analyzed based on demographic comparisons. Phase 2b used the datasets collected during Phase 2a.

### Phase 1: Translation and Cultural Adaptation of the eHLQ

#### Overview

The Phase 1 study was performed following Translation Integrity Procedure (TIP; version 5), which was developed by Hawkins and Osborne [43]. TIP ensures cultural and linguistic appropriateness for the target audience and natural language and readability for those with low literacy levels and demonstrates equivalent measurement performance to the original source version [43]. A bilingual translator (YM) created the initial Japanese draft using a “translation management grid and item intents” provided by Swinburne University of Technology, the licensor of the eHLQ. The item intents described in the translation management grid were carefully considered during translation. The draft was then reviewed by a second bilingual translator (RS) for improvements. After revisions, the translators met to discuss linguistic and cultural equivalency between the English and Japanese versions. Upon agreement, a native English-speaking bilingual translator (MN) blinded to the original backtranslated the Japanese version.

#### Consensus Meetings and Cognitive Interviews

The forward and backward translations were sent to the original eHLQ developers for feedback. An online consensus meeting with the original eHLQ developers, held in February 2022, involved 7 experts in fields including digital health, public health, nursing, physiotherapy, medical education, pharmacy, and sociology. Each item was reviewed to ensure it aligned with the original context and intent. Attention was also paid to maintaining the versatile vocabulary used in the original eHLQ to ensure its longevity. Language consistency and item intent were verified, and revisions were made accordingly.

Cognitive interviews followed, using the consensus-approved version, to assess participant comprehension of the instructions, response format, and item content. Participants were recruited through personal contacts and their extended networks, purposefully selecting individuals diverse in region, degree of urbanization, age, gender, and education to minimize sampling bias. To maintain consistency, the interviews were performed by the first author. Participants received a 1000 JPY (equivalent to approximately US $6.50) gift card and reimbursement for any transportation costs. They completed interviews in person or online, reading each item aloud to ensure all terms and *Kanji* (Chinese characters used in Japanese writing) could be comprehended. To determine their understanding of cognitive factors underlying responses, participants were asked (in Japanese), “What were you thinking when you answered that question?” The following question was then asked (in Japanese) if needed: “Why did you select that response option?” The interviewer carefully took notes and confirmed participants’ comments on every item before moving to the next one. The interviews were also recorded with participants’ consent. Participants’ responses and comments were grouped by item and tabulated using an online spreadsheet platform, and the data were shared with the coauthors for review. Based on participant responses, if deemed necessary, the translations were adjusted to fit the items as intended. The final version after any adjustments following the interviews was shared with the original developers, and a final consensus meeting was held in April 2023.

### Phase 2a: Psychometric Testing

#### Overview

The Japanese version of the eHLQ was administered to a large demographically representative sample to evaluate its psychometric properties. The Research Electronic Data Capture platform, developed at Vanderbilt University in the United States [44,45], was used for the survey. The questionnaire consisted of 35 eHLQ items, displayed with a maximum of 10 items per screen, demographic questions, questions about the frequency of information and communication technology (ICT) use, and questions about health status.

#### Recruitment

Participants were recruited through an online survey panel operated by a Japanese survey company (ASMARQ Co., Ltd., Tokyo, Japan) to ensure broad demographic representation, with screening criteria based on age, sex, location, and education. After screening, participants were directed to the main questionnaire online through Research Electronic Data Capture. Adults aged 18 years and older were eligible. Rather than recruiting only current older adults, we recruited the entire adult age range because aging societies encompass populations of all ages. Furthermore, the following were considered: (1) while the instrument was intended for long-term use, younger adults would age into the target cohort, and (2) older adults often rely on younger family members for help using digital health services, making younger adults relevant as well. The Ministry of Health, Labor, and Welfare, and Statistics Bureau of Japan defined those aged ≥65 years as elderly (comprising 36.1 million, 28.7% of the population) [46,47]. The participants were intentionally recruited from age groups in proportions mirroring the general population. Participants who completed the survey received points from the survey company. These points could be exchanged for a gift certificate worth approximately US $2. The web-based survey was conducted between July 3 and 17, 2023. At the time, internet users included 82.1%, 56.2%, and 26.4% of those aged in their 60s, 70s, and ≥80s, respectively [48]. Therefore, an in-person survey was conducted for those aged ≥65 years. Potential respondents were initially approached at hospitals affiliated with the authors’ institution. However, due to COVID-19 restrictions, an insufficient number of participants were available from this source, so participants were also recruited via “*Silver Jinzai Center*” facilities for older adults, which are nonprofit organizations in multiple regions of Japan that provide work for senior citizens in local communities. Participants received 2000 JPY (approximately US $13), including transportation fees, in line with the typical rate paid by the human resource center. The first author conducted in-person, face-to-face interviews with these participants and administered the eHLQ and the same demographic questions given to participants in the online survey. In total, a sample of over 500 participants was targeted, which was considered adequate for the measurement properties analyses [49].

#### Classical Test Theory for Construct Validity

Confirmatory factor analysis was performed using Mplus Version 8.1 (Muthén & Muthén, Los Angeles, CA, USA), with 1-factor and 7-factor models for the scales. Since eHLQ scores are categorical, we used weighted least squares mean and variance estimators, which are robust and suitable for estimating categorical data [50]. The comparative fit index (CFI), standardized root mean residual (SRMR), and standardized expected parameter change (SEPC) were obtained with Mplus analysis using the MODINDICES (0) output option, with CFI >0.95 [51] and SRMR ≤0.08 [52], indicating acceptable model fit.

#### Item Response Theory

IRT, which is a statistical framework for comparing test versions using a standardized metric [53], was applied to analyze item location and discrimination using Mplus Version 8.1 (Muthén & Muthén, Los Angeles, CA, USA). Boundary characteristic curves were plotted in Stata SE 18.0 (StataCorp, College Station, TX, USA) to visualize item difficulty, representing the probability of responses at various difficulty levels [54].

### Phase 2b: Descriptive Analysis

#### Score Analysis by Demographic Characteristics

Group differences impacting eHLQ scores were analyzed using ANOVA (version 29.0; SPSS , IBM, USA). *P* values less than .05 indicated statistical significance. Multiple comparisons were conducted using post hoc tests in SPSS with the Bonferroni correction. For 2-group comparisons, the independent *t* test was performed using SPSS. Two-sample *t* tests were conducted using 2-sided *P* values, with the equal variances assumption based on Levene test results (*P*≥.05 = equal variances assumed; *P*<.05 = equal variances not assumed).

Cohen *d* was used to quantify effect sizes, calculated as *d*=(M₁–M₂) ⁄ SD_pooled_. Effect sizes were interpreted as medium (0.50 ≤ *d* <0.80) and large (*d*≥0.80), both of which were considered worth discussing. Effect sizes with Cohen *d* below 0.50 were considered small.

#### Permission for Translation

Translation of the eHLQ to other languages requires a translation license. The authors obtained permission from Swinburne University of Technology, which manages the license. The authors also obtained permission from Prof. Lars Kayser, the corresponding author of the original eHLQ manuscript [18].

### Ethical Considerations

The Institutional Review Board of Juntendo University Faculty of Health Science reviewed and granted approval (Approval No. 22‐015). For the face-to-face version of the survey, including both cognitive interviews and in-person surveys for psychometric analysis, participants received a printed information sheet. Participants gave written consent by signing the form. For the online survey, each prospective participant read an electronic information sheet before beginning the online questionnaire. Participants provided consent by clicking the “I agree” button. The survey could not be accessed without this affirmative action. Participants received modest incentives, which were described earlier. For privacy and confidentiality protection, no direct identifiers were collected. Participants were automatically given a random study ID, and the response file contained only this ID and the survey answers. The deidentified dataset was stored on a password-protected hard drive, which was stored in a locked cabinet in a building requiring a security card for entry.

## Results

### Phase 1: Translation and Cultural Adaptation of the eHLQ

#### Initial Translation and Consensus Meeting

The initial translation was conducted with a focus on clarity and naturalness of expression. Consequently, for items containing the term “technology,” the type of technology was sometimes specified, such as “medical digital devices” or “online services,” depending on the context, to reduce vagueness and confusion. Prior to the consensus meeting, the Japanese translation and back translation were sent to the Danish eHLQ developer team for review and feedback.

During the meeting, each individual translated item was discussed to confirm that it was a faithful representation of the intended meaning of the original version. The discussion included selection of semantically appropriate Japanese vocabulary (eg, correspond vs adapt), a level of difficulty (eg, know vs be able to), and item intent (eg, “experience,” not “belief”). The Danish team explained that versatile vocabularies were intentionally chosen for longevity of use; therefore, as long as these made sense, the translation should be as close as possible to the original version. Accordingly, “medical digital devices” and “online services” were reverted to “technology.” Cultural adaptation was also considered while maintaining the intended meaning of each item. For example, in the item regarding participant data-sharing method, the Japanese version added the term “mainly” to indicate it does not mean “definitively always,” addressing the tendency of Japanese people to hesitate to clearly state abilities or preferences. In addition, words requiring confirmation during the cognitive interviews were listed. For example, the phrase “measurement about my body,” which was considered somewhat awkward, was confirmed to be checked in a subsequent cognitive interview to ensure it would be correctly understood. The revised version was developed after the consensus meeting, shared with the Danish team, and subsequently approved for use in the cognitive interviews.

#### Cognitive Interviews

A total of 12 people participated in the cognitive interviews, comprising 6 males and 6 females aged 19-77 years with diverse educational backgrounds and from diverse locations. The participants’ ages were 19, 29, 40, 65, 69, and 71 years for men and 23, 38, 40, 56, 62, and 77 years for women. A total of 6 interviews were conducted in person face-to-face, while the remaining 6 were performed online. Participants were from 9 different prefectures among the 7 regions of Japan, including 2 from remote islands. While most participants pointed out that some words or terms were unclear, 4 terms or phrases were frequently discussed.

1. eHealth system: The term “eHealth” was relatively new in Japan, so we initially translated it as “digital health system.” However, participants found it difficult to understand. Since “eHealth” was a novel term, participants gave close attention to it when it appeared in the instructions. Based on this feedback, we chose to use “eHealth system” (eヘルス・システム) in the Japanese version.
2. Technology: Initially, “technology” was translated directly, using the Japanese pronunciation. Many participants associated it with advanced medical technology, such as computed tomography and magnetic resonance imaging scans, rather than everyday digital technology, such as smartphones, internet services, home-use health devices, and so forth. To more accurately convey the concept, we replaced the term with “digital gijutsu” (デジタル技術), which translates to digital technology.
3. Best for me: In the English version of the eHLQ, this phrase means health care most suitable for the participant. It was first translated as “*pittarina*” (ぴったりな), a common colloquial term for “fits perfectly.” However, some participants found this vague, so we changed it to “*saiteki*” (最適), a more formal term meaning “best.”
4. My individual needs: Some participants questioned the meaning of this phrase, most likely because there is no direct equivalent in Japanese. After extensive discussions among the original eHLQ developers and Japanese translators, “*kitai*” (期待), meaning “expectations,” was adopted.

In contrast, the literal translation of “measurements about my body (自分の身体の測定値)” was initially thought to be difficult to understand because the combined use of the Japanese words “measurements” and “my body” sound somewhat awkward. However, all 12 participants understood it well. Therefore, this was not revised. The final Japanese version of the eHLQ can be found in Multimedia Appendix 1.

### Phase 2a: Psychometric Testing

#### Demographics and Digital Health Literacy Scores

**Figure 2.**
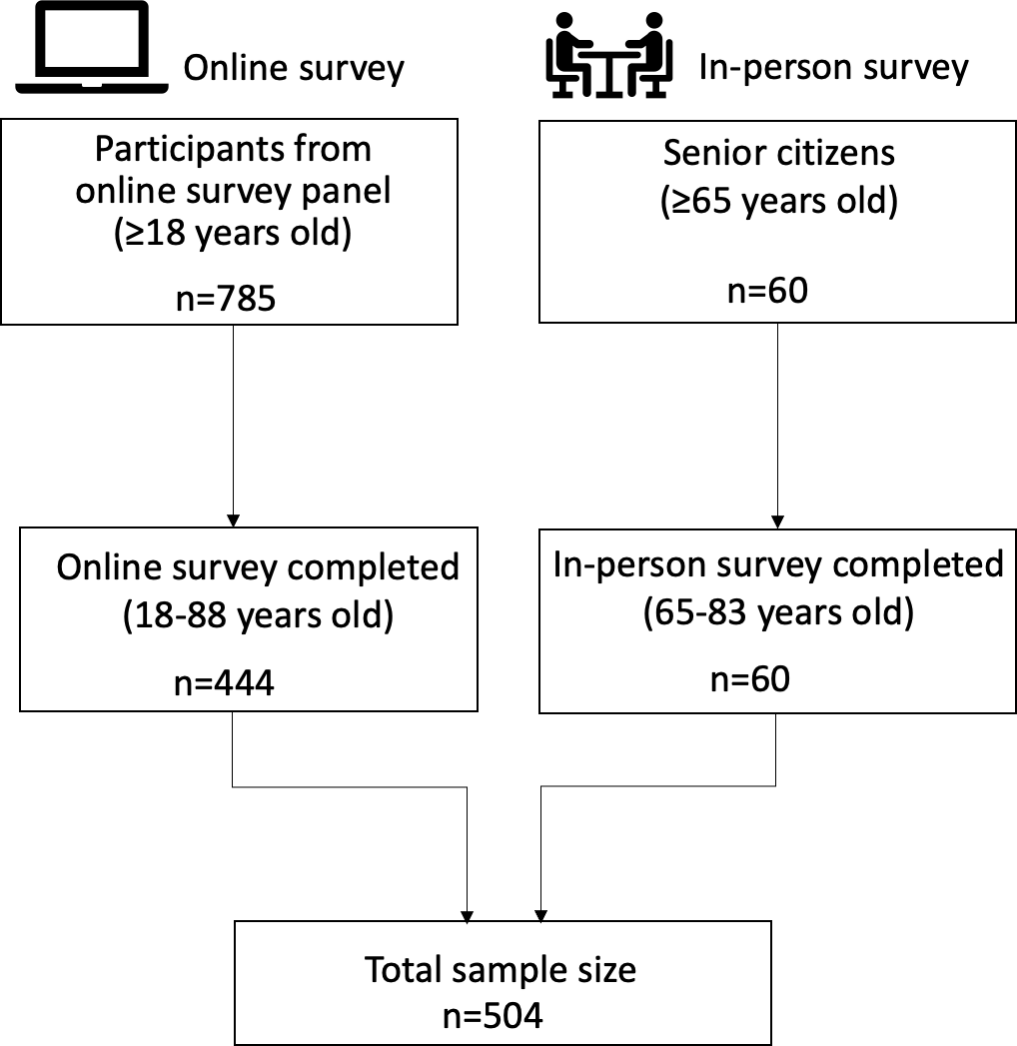
Participant recruitment flow for psychometric testing of the Japanese version of the eHealth Literacy Questionnaire (eHLQ).

Of the 785 participants who responded to the online survey, 444 completed the questionnaire. An additional 60 participants completed personal face-to-face interviews, yielding a total sample size of 504. Their mean age was 51.6 years (range 18‐88, SD 17.5), with 159 participants (31.5%) aged ≥65 years. Gender distribution included 257 (51.0%) male, 245 (48.6%) female, and 2 (0.4%) other. The participant recruitment flowchart is shown in Figure 2, and participant demographics are summarized in Table 1.

**Table 1.** Interview method and demographic variables of participants (n=504).

Characteristics	Participants, n (%)	
Method (age range, y)		
Online (18‐88)	444 (88.1)	
In person face-to-face (65‐83)	60 (11.9)	
Age (mean 51.6, SD 17.5)		
18‐19 years	8 (1.6)	
20s	72 (14.3)	
30s	75 (14.9)	
40s	77 (15.3)	
50s	74 (14.7)	
60s	96 (19.0)	
70s	94 (18.7)	
80s	8 (1.6)	
≥65 years	159 (31.5)	
Gender		
Male	257 (51.0)	
Female	245 (48.6)	
Other (“nonbinary” and “other”)	2 (0.4)	
Region of residence in Japan		
Hokkaido (northernmost island)	22 (4.4)	
Tohoku (northeast)	46 (9.1)	
Kanto (includes Tokyo)	227 (45.0)	
Chubu (central region)	53 (10.5)	
Kinki (west central region)	73 (14.5)	
Chugoku and Shikoku (western region)	52 (10.3)	
Kyushu and Okinawa (southern region)	31 (6.2)	
Degree of urbanization		
Special wards (Tokyo’s 23 wards)	83 (16.5)	
Ordinance-designated city	139 (27.6)	
City	259 (51.4)	
Town or village	23 (4.6)	
Education		
Junior high school (ISCED^a^ level 2)	10 (2.0)	
High school (ISCED level 3)	153 (30.4)	
Vocational school (ISCED level 5)	50 (9.9)	
Junior college (ISCED level 5)	49 (9.7)	
Technical college (ISCED level 5)	1 (0.2)	
University (bachelor’s degree) (ISCED level 6)	216 (42.9)	
Graduate school (master’s degree) (ISCED level 7)	19 (3.8)	
Graduate school (doctoral degree) (ISCED level 8)	6 (1.2)	
Working hours per week		
Unemployed or retired	151 (30.0)	
Less than 20 hours	67 (13.3)	
20‐39 hours	52 (10.3)	
Full-time (40+ h)	189 (37.5)	
Working hours vary widely from week to week	20 (4.0)	
Other than above (eg, student)	22 (4.4)	
Unknown	3 (0.6)	
Type of ICT^b^ use (at least once a week)		
Internet services^c^	471 (93.5)	
Digital health services via internet^d^	125 (24.8)	
Social network services^e^	383 (76.0)	
Computer^f^	390 (77.4)	
Smartphone	450 (89.3)	
Mobile phone other than smartphone	39 (7.7)	
Tablet computer device^g^	112 (22.2)	
Internet access via TV	112 (22.2)	
Home game consoles^h^	93 (18.5)	
Other	43 (8.5)	
Self-rated health status		
Very good	40 (7.9)	
Good	137 (27.2)	
Normal	257 (51.0)	
Bad	65 (12.9)	
Very bad	5 (1.0)	

a ISCED: International Standard Classification of Education.

b ICT: information and communication technology.

c Examples given to the participants: internet browsing, searching, shopping, using email, and so forth.

d Examples given to the participants: booking appointments for a clinic, searching for medical information, using health apps on smartphones, and so forth.

e Examples given to the participants: Facebook, LINE, Twitter (X), mixi, Instagram, and so forth.

f Examples given to the participants: desktop computer, laptop computer.

g Examples given to the participants: iPad, E-reader, and so forth.

h Examples given to the participants: PlayStation, and so forth.

The mean eHLQ scores of the 7 scales ranged from 2.72 to 2.30. Participants reported the highest scores on item 35 in scale 5 (Motivated to engage with digital services) and the lowest on item 16 in scale 6 (Access to digital services that work). The summary of the results is shown in Table 2.

**Table 2.** Descriptive and psychometric properties of the 7 scales of the Japanese version of the eHealth Literacy Questionnaire (eHLQ; n=504).

Scales	Mean (SD)		Score range	Cronbach *α*	CFI^a^	SRMR^b^	SEPC^c^
1^d^	2.47 (0.57)		2.24 (Q25)-2.68 (Q13)	0.85	0.99	0.02	0.13
2^e^	2.55 (0.49)		2.26 (Q12)-2.80 (Q15)	0.78	0.96	0.04	0.23
3^f^	2.40 (0.58)		2.30 (Q32)-2.47 (Q08)	0.84	0.97	0.04	0.21
4^g^	2.54 (0.51)		2.17 (Q14)-2.72 (Q01)	0.81	0.99	0.03	0.17
5^h^	2.72 (0.52)		2.60 (Q02)-2.81 (Q35)	0.85	1.00	0.01	−0.06
6^i^	2.30 (0.53)		2.05 (Q16)-2.56 (Q03)	0.84	0.99	0.03	0.17
7^j^	2.40 (0.56)		2.29 (Q18)-2.48 (Q33)	0.85	1.00	.01	−.07

a CFI: comparative fit index (>0.95).

b SRMR: standardized root mean residual (≤0.08).

c SEPC: standardized expected parameter change (>0.25 indicating misspecification).

d Scale 1: Using technology to process health information.

e Scale 2: Understanding of health concepts and language.

f Scale 3: Ability to actively engage with digital services.

g Scale 4: Feel safe and in control.

h Scale 5: Motivated to engage with digital services.

i Scale 6: Access to digital services that work.

j Scale 7: Digital services that suit individual needs.

#### Reliability

Internal consistency determined using Cronbach *α* exceeded 0.80 for all scales except for scale 2 (Understanding of health concepts and language), which scored 0.78, indicating reliability from acceptable to good (Table 2).

#### Construct Validity

A 1-factor CFA analysis showed good fit for the Japanese eHLQ across all scales based on CFI (≥0.96) and SRMR (≤0.04) values (Table 2). All items had significant factor loadings (≥0.50) (Multimedia Appendix 2), with SEPC values for all 7 scales being <0.25, and 5 of these scales having SEPC values <0.20 (Table 2). Interfactor correlations were analyzed using a 7-factor model, showing a suitable range of 0.26-0.59. The model diagram is shown in Multimedia Appendix 3.

#### Item Response Theory

IRT analysis demonstrated that estimated item locations were generally well distributed, except for items 6 and 8 in scale 3 (Ability to actively engage with digital services), and items 24 and 35 in scale 5 (Motivated to engage with digital services). Item discrimination values were all >0, being from 1.03 to 3.72, with the narrowest and widest range noted for item 14 (0.74‐1.31) and item 31 (2.73‐4.71) (Multimedia Appendix 4). Boundary characteristic curves indicated difficulty parameters around 0.5 on the latent trait scale, with slope steepness showing good item fit (Multimedia Appendix 5).

### Phase 2b: Descriptive Analysis

#### Demographic Group Comparisons of the eHLQ Scores

Participants were grouped by demographic characteristics for further analysis, essentially between-groups comparisons. Regional and gender classifications showed differences in 1 scale each; however, post hoc analysis did not identify any specific group differences. Degree of urbanization and education level showed differences in 2 scales; these were also observed in the post hoc analysis, though effect sizes were small. The working hour classification showed differences in 4 scales, with the effect size of scale 6 (Access to digital services that work) being >0.75. The comparison showing large effect size was between “unemployed or retired” and “other than above (eg, student),” and that of medium effect size was between “20‐39 hours per week” and “other than above (eg, student),” with the “other than above (eg, student)” group having higher mean scores. Age groups in 10-year ranges showed differences in all but scale 2 (Understanding of health concepts and language); post hoc analysis confirmed these findings, with 5 scales showing effect sizes considered worth discussing. The most frequently observed comparisons showing medium or large effect sizes were between the age 20s and 60s groups (3 scales) and between the age 50s and 70s groups (2 scales), with the 20s and 70s groups having higher mean scores. Differences in eHLQ scores by self-reported health status were observed across all 7 scales, with medium effect sizes found in the comparisons between “‘very good or good” and “bad or very bad” in 5 of the 7 scales. The group comparisons of eHLQ scores across demographic variables, the results of post hoc analysis, point estimates with 95% CIs, and effect sizes are presented in Multimedia Appendix 6.

Two group comparisons revealed that those aged ≥65 years scored higher on 3 scales, compared to those aged <65 years; however, the effect sizes were all small. Individuals who reported that they used the internet at least once a week scored higher on scales 1 and 3, which both related to information and media literacy, with scale 3 showing a medium effect size. In contrast, differences were found across all 7 scales, with medium effect sizes observed among people who used digital health services at least once a week compared to those who used them less frequently. Participants with chronic disease(s) scored higher on 3 scales, but those effect sizes were small. The results of the 2-group comparisons are summarized in Table 3.

**Table 3.** Two-group comparisons of the Japanese version of the eHealth Literacy Questionnaire (eHLQ) scores across various demographics (n=504).

	eHLQ scores						
Scale	1^a^	2^b^	3^c^	4^d^	5^e^	6^f^	7^g^
Age (y)							
<65 (n=345)	2.49	2.51	2.44	2.50	2.67	2.31	2.40
≥65 (n=159)	2.42	2.65	2.33	2.64	2.83	2.28	2.42
Mean difference	0.07	−0.14	0.10	−0.13	−0.15	0.03	−0.02
95% CI lower	−0.04	−0.23	−0.01	−0.22	−0.24	−0.07	−0.12
95% CI upper	0.18	−0.01	0.21	−0.05	−0.06	0.12	0.08
*P* value	.20	<.01	.07	<.01	<.01	.59	.68
Levene test (sig.)	.60	<.01	.21	<.01	<.01	<.01	.03
Effect size	0.12	0.30	0.18	0.26	0.30	0.05	0.04
ICT^h^ use (internet)							
At least once a week (n=471)	2.48	2.57	2.42	2.55	2.73	2.30	2.41
Less than once a week (n=33)	2.26	2.41	2.12	2.47	2.61	2.26	2.38
Mean difference	0.22	0.15	0.30	0.08	0.12	0.05	0.03
95% CI lower	0.02	−0.08	0.10	−0.10	−0.07	−0.14	−0.17
95% CI upper	0.42	0.39	0.51	0.27	0.30	0.24	0.23
*P* value	.03	.20	<.01	.37	.22	.63	.79
Levene test (sig.)	.13	.02	.34	.08	.19	.69	.76
Effect size	0.39	0.31	0.53	0.16	0.22	0.09	0.05
ICT use (digital health services)							
At least once a week (n=125)	2.76	2.79	2.64	2.78	2.95	2.57	2.69
Less than once a week (n=379)	2.37	2.48	2.33	2.47	2.64	2.21	2.31
Mean difference	0.39	0.31	0.31	0.31	0.31	0.36	0.38
95% CI lower	0.28	0.22	0.20	0.21	0.21	0.26	0.27
95% CI upper	0.51	0.41	0.43	0.41	0.41	0.47	0.49
*P* value	<.01	<.01	<.01	<.01	<.01	<.01	<.01
Levene test (sig.)	.04	.17	.09	<.01	.06	.59	.16
Effect size	0.72	0.66	0.55	0.63	0.61	0.71	0.71
Chronic diseaswe							
With chronic disease(s) (n=194)	2.44	2.62	2.39	2.64	2.78	2.32	2.43
No chronic disease (n=310)	2.48	2.51	2.41	2.48	2.68	2.29	2.39
Mean difference	−0.04	0.10	−0.02	0.16	0.10	0.03	0.05
95% CI lower	−0.14	0.02	−0.12	0.07	0.01	−0.07	−0.05
95% CI upper	0.06	0.19	0.09	0.25	0.19	0.12	0.15
*P* value	.44	.02	.78	<.01	.03	.59	.36
Levene test (sig.)	.54	.10	.79	.02	.21	.07	.63
Effect size	0.07	0.21	0.03	0.31	0.20	0.05	0.08

a Scale 1: Using technology to process health information.

b Scale 2: Understanding of health concepts and language.

c Scale 3: Ability to actively engage with digital services.

d Scale 4: Feel safe and in control.

e Scale 5: Motivated to engage with digital services.

f Scale 6: Access to digital services that work.

g Scale 7: Digital services that suit individual needs.

h ICT: information and communication technology.

## Discussion

### Principal Results

The Japanese eHLQ was translated from the English version, and its reliability was assessed through psychometric analysis. Data collected from a representative sample of Japan aged from 18 to 88 years were analyzed using classical test theory, IRT, and comparative statistical methodologies. The results indicated the Japanese eHLQ has strong-to-acceptable measurement reliability. Comparative analyses of demographic factors revealed that scores across all 7 scales differed among groups classified by self-reported health status and between groups classified by frequency of digital health service use. Age groups showed differences on 6 scales; however, 2-group comparisons (≥65 y vs <65 y) revealed the elderly scored higher on scales 2, 4, and 5, albeit the effect sizes were small.

### Psychometric Analysis

Classical test theory and IRT analyses indicated the instrument was satisfactory or acceptable. All 35 items exhibited standardized loadings above 0.50, indicating that each item strongly represents its respective scale. A potential concern is that the eHLQ has some substantial interfactor correlations [18,34], which may be related to the high factor loadings. According to Kayser et al [18], those correlations are likely caused by the scales sharing the same causal pathway while measuring different constructs. Since content differentiation among the scales has been theoretically supported [18,34,37], this is unlikely to compromise the interpretation of the scale scores.

Regarding IRT analysis, items 6 and 8 on scale 3 were less well distributed compared to other items. These 2 items assess different levels of difficulty regarding the ability to engage with digital services. While item 6 assesses general knowledge of digital technology, item 8 evaluates practical performance ability with the technology. The results indicate that among Japanese participants, those with knowledge of digital technology overlapped with those who could use the technology. Other poorly distributed items included 24 and 35 on scale 5. These items assess motivation to engage with digital services and evaluate expectations of digital technologies, namely one for receiving services and the other for utilizing them. Since scores for scale 5 were generally high, this result may reflect characteristics of Japanese people.

Among the top 3 items with the highest item locations (items 14, 16, and 29), items 16 and 29 focus on digital health services that are either unavailable or have very limited availability in Japan, making them challenging. Item 14 examines the acquisition of advanced understanding sufficient to utilize health data in health care settings, which may have made participants reluctant to respond “agree” or “strongly agree.” Given that the eHLQ contains items with different difficulty levels, item 14 may help distinguish participants in greater detail. Apart from these items, the IRT analysis showed well-distributed responses.

### Relationship Between eHLQ Scores and Participant Demographic Factors

Differences in eHLQ scores were examined across demographic variables. Several studies have reported that education level is associated with both ICT use and digital health literacy [55-57]. In this study, score differences were observed between the education level groups on some scales, particularly scales 1, 2, and 3, which examine participants’ skills and knowledge, and scale 6, which is associated with participants’ experiences with digital health services. However, the differences were minimal as indicated by small effect sizes. Furthermore, scores on scales 4, 5, and 7, which examine participants’ beliefs, motivations, perceptions, and expectations regarding digital health services, showed no differences. This partial effect of education level on eHLQ scores in Japan differs from findings in Taiwan and Serbia, where education level affected all 7 scales [31,58].

Analysis of internet use frequency and eHLQ scores revealed that individuals who used the internet at least once a week scored higher on 2 scales (Table 3), with the effect size for scale 3 (Ability to actively engage with digital services) being medium. However, since 93.5% (471/504) of the participants reported using internet services at least once a week, internet use alone may not be a reliable indicator of digital health literacy. In contrast, differences were observed across all 7 scales with medium effect sizes when comparing participants by their frequency of digital health service use. Since using digital health services can enhance digital health literacy [9], these differences may become more significant over time.

Age is a known predictor of digital health literacy [31,59], which was also observed in this study (Multimedia Appendix 6). However, when dividing participants into 2 age groups, the analysis revealed that differences between those under and those over 65 years old showed small effect sizes across all 7 scales (Table 3). These results may have been influenced by the multifaceted nature of the eHLQ assessment tool. Using an instrument that emphasizes internet operating skills and technological knowledge might yield different results. Additionally, older adults in Japan lived through Japan’s period of rapid economic growth, during which they witnessed remarkable technological advancements. As a result, even if their personal digital skills are limited, they may still hold positive attitudes toward digital technology.

Those who rated their health status as “very good” or “good” scored higher on all 7 scales, with 5 scales showing medium effect sizes. This result is consistent with a previous eHLQ study [31]. Interestingly, the 2-group comparison between participants with and without chronic disease(s) did not show notable differences. This suggests that self-reported health status was more important than actual disease status in relation to eHLQ scores (Table 3 and Multimedia Appendix 6).

### Implications for Practice: Digital Health Services for Japan’s Super-Aged Society

Although implementation was not within the scope of this study, the following considerations may inform health care workers, system developers, and policy makers, as well as future research development.

Neither being over 65 years old nor having chronic disease(s) was linked to low eHLQ scores. Self-management has been proven as a strategy for chronic diseases care [60], and several digital tools, such as apps, wearable devices, and remote monitoring systems, are available for this purpose [60]. Since digital health services are recommended for self-management of NCDs, or chronic diseases [28,29], employing these technologies for patients with chronic disease(s) may help alleviate the burden on the overloaded Japanese health care system [61].

There are some potential risks to consider when promoting digital health services in Japan. Comparing 10-year age groups, participants in their 50s and 60s tended to score lower than other age groups (Multimedia Appendix 6). Despite being relatively familiar with the internet [48], these groups may still need support in using digital health services. While older adults often rely on younger family members for assistance in using digital services—a tendency that was also frequently noted during face-to-face interviews—these supporting generations may not always be able to provide adequate help. Another concern is the perception of security and safety in digital technology, assessed using scale 4 (feel safe and in control). Agreeing or strongly agreeing with those items would typically require some ICT knowledge. However, this result warrants caution, especially for people who scored well or average on scale 4 despite limited ICT usage. During face-to-face interviews with elderly people, the author (YM) observed participants often mumbling phrases like “It’s supposed to be” or “I want to believe so” while responding to items regarding security and safety. This could be due to Japanese cultural traits, such as hierarchical and conformist tendencies, which may inhibit critical thinking, so high scores on those items may be unreliable measures of understanding internet security [62]. Nevertheless, while a high score on scale 4 should not be seen as a barrier to facilitating digital health services, health care workers should be aware that users with high scores on scale 4 may still be vulnerable to internet security risks and not openly express concerns.

This study revealed that people who used digital health services at least once a week had higher digital health literacy. IRT analysis demonstrated response scores of individuals who reported technological knowledge overlapped with those who reported capability in using technology. This factor warrants caution, as current systems may be tailored to users with sufficient digital health literacy and may be unsuitable for those who do not regularly use these services. Developers of digital health services should aim to avoid complexity that requires high digital health literacy. Instead, technology should be designed to accommodate user expectations and compensate for gaps in skills, knowledge, or user experience—areas that can be assessed using the eHLQ. Due to advancements in information and communication technologies, required digital health literacy is rapidly changing. Since we took great care to maintain the versatile vocabulary that eHLQ uses to ensure its longevity, the instrument is expected to help monitor digital health literacy in Japan in the coming years.

### Limitations

The survey excluded income level, since asking about income is considered impolite in Japanese culture [63]. Income questions might create a barrier between participants and researcher, particularly in face-to-face interviews. Among the 159 participants aged ≥65 years old, 99 (62.3%) participated online. To do so, they had previously registered with the survey platform, meaning they likely had better access to ICT than others in the same age group. For the face-to-face survey, participants recruited through human resource centers for older adults may have had less cognitive impairment and better overall health status compared to average for their age group. Further investigation of older adults who require physical and cognitive support is necessary for a more comprehensive understanding of the impact of age on digital health literacy in Japan. Although the current university enrollment rate in Japan is 57.7%, the population in this study may have held a higher education level than the general Japanese population, with 47.8% (241/504) of the participants having International Standard Classification of Education levels >6.

### Conclusions

Psychometric analysis showed that the Japanese version of the eHLQ is likely a reliable and effective tool for assessing digital health literacy in Japan. There were no notable differences between scores of those aged above and below 65 years, or those with and without chronic disease(s), as indicated by small effect sizes. Service providers should be aware of users’ digital health literacy—including skills, knowledge, expectations, and perceptions—as assessing these aspects is important for effectively promoting such services. The Japanese version of the eHLQ is well suited for assessing digital health literacy and is expected to be used to monitor this literacy and identify additional support needs, thereby potentially contributing to the health care system in Japan.

## Supplementary material

10.2196/68529 Multimedia Appendix 1 eHealth Literacy Questionnaire (eHLQ) in English and Japanese translation.

10.2196/68529 Multimedia Appendix 2 Standardized factor loadings of the 1-factor and 7-factor models of the Japanese version of the eHealth Literacy Questionnaire (eHLQ).

10.2196/68529 Multimedia Appendix 3 A confirmatory factor analysis of the Japanese version of eHLQ (a 7-factor model).

10.2196/68529 Multimedia Appendix 4 Item response theory (IRT) analysis of the Japanese version of the eHealth Literacy Questionnaire (eHLQ).

10.2196/68529 Multimedia Appendix 5 Boundary characteristic curves of the 35 items of the Japanese version eHealth Literacy Questionnaire (eHLQ).

10.2196/68529 Multimedia Appendix 6 Descriptive and comparative statistics for the Japanese version eHealth Literacy Questionnaire (eHLQ) scores across demographic groups.

## Abbreviations

CFI: comparative fit index
eHLF: eHealth Literacy Framework
eHLQ: eHealth Literacy Questionnaire
ICT: information and communication technology
IRT: item response theory
NCDs: Noncommunicable diseases
SEPC: Standardized Expected Parameter Change
SRMR: Standardized Root Mean Residual
TIP: Translation Integrity Procedure
WHO: World Health Organization
