# Supplementary material for: Translation, Cultural Adaptation, and Validation of the Japanese eHealth Literacy Questionnaire Among Users in a Super-Aged Society: Mixed Methods Study
Source: J Med Internet Res. 2025 Nov 26;27:e68529. doi: 10.2196/68529 (PMC12661597; doi:10.2196/68529)
Supplement: Multimedia Appendix 1 [file jmir-v27-e68529-s001.pdf]

Multimedia Appendix 1. eHealth Literacy Questionnaire (eHLQ) in English and Japanese translation.

		Scale/Item	Japanese translation
		**1. Using technology to process health information**	
1	Q7	I use technology to find…	私はデジタル技術を使って...
2	Q11	I often use technology…	私は自分の健康問題を…
3	Q13	Technology helps me…	デジタル技術は自分に…
4	Q20	I use technology to share…	自分の体調に関する…
5	Q25	I use technology to organize…	私はデジタル技術を使って...
		**2. Understanding of health concepts and language**	
6	Q5	The knowledge I have helps me…	私の知識は…
7	Q12	I have enough information…	私は自分の…
8	Q15	I understand medical results…	私は自分の診察の…
9	Q21	Overall, I understand how…	私は自分の体の…
10	Q26	I use measurements about…	私は自分の体調を…
		**3. Ability to actively engage with digital services**	
11	Q4	I know how to use technology…	私はデジタル技術を使って...
12	Q6	I know how to make…	自分にとって便利な…
13	Q8	I can enter data into…	私は健康に関する…
14	Q17	I quickly learn how to find…	私は新しいデジタル技術に…
15	Q32	I easily learn to use new…	私は健康に関する…
		**4. Feel safe and in control**	
16	Q1	I am sure that my health data…	私の医療情報は…
17	Q10	My electronic healthcare data…	私の電子化された医療情報は…
18	Q14	I have a clear understanding…	医療従事者が私の…
19	Q22	I am sure that only authorized…	規則で許可された人のみが…
20	Q30	I am confident that healthcare…	医療従事者は私の…
		**5. Motivated to engage with digital services**	
21	Q2	Technology makes me…	デジタル技術のおかげで、
22	Q19	I find technology helps me…	デジタル技術は自分自身の
23	Q24	I find I get better services…	デジタル技術を活用すると…
24	Q27	Technology improves…	デジタル技術は私と…
25	Q35	I find technology useful…	デジタル技術は私の…
		**6. Access to digital services that work**	
26	Q3	Information about my health…	私の医療情報は…
27	Q9	My healthcare providers…	私の医療従事者は…
28	Q16	My health data are available…	私は自分の医療情報に…
29	Q23	All the health technology I use…	私が利用している健康関連の…
30	Q29	Most of my healthcare…	私を担当しているほとんどの…
31	Q34	I have access to health…	私がいつでも利用できる…
		**7. Digital services that suit individual needs**	
32	Q18	I find that eHealth systems…	eヘルス・システムは、私の…
33	Q28	I find eHealth systems seem to…	eヘルス・システムは、私の…
34	Q31	I find eHealth systems are…	eヘルス・システムは、私に…
35	Q33	eHealth systems provide me…	eヘルス・システムは、私が…

*Items are truncated. For the full list of items, please contact the eHLQ Licensing Officer from Swinburne University of Technology.
