# Supplementary material for: Translation, Cultural Adaptation, and Validation of the Japanese eHealth Literacy Questionnaire Among Users in a Super-Aged Society: Mixed Methods Study
Source: J Med Internet Res. 2025 Nov 26;27:e68529. doi: 10.2196/68529 (PMC12661597; doi:10.2196/68529)
Supplement: Multimedia Appendix 2 [file jmir-v27-e68529-s002.pdf]

Multimedia Appendix 2. Standardized factor loadings of the 1-factor and 7-factor models of the Japanese version of the eHealth Literacy Questionnaire(eHLQ).

Scale	Item	1-Factor loading	95% CI	7-Factor loading	95% CI
1. Using technology to process health information	eHLQ7	0.79	(0.75 – 0.83)	0.78	(0.75 – 0.81)
	eHLQ11	0.82	(0.78 – 0.86)	0.75	(0.71 – 0.78)
	eHLQ13	0.76	(0.72 – 0.81)	0.82	(0.80 – 0.85)
	eHLQ20	0.86	(0.82 – 0.89)	0.82	(0.80 – 0.85)
	eHLQ25	0.78	(0.73 – 0.82)	0.84	(0.81 – 0.86)
2. Understanding of health concepts and language	eHLQ5	0.74	(0.68 – 0.79)	0.75	(0.71 – 0.79)
	eHLQ12	0.80	(0.75 – 0.84)	0.75	(0.72 – 0.79)
	eHLQ15	0.72	(0.66 – 0.77)	0.67	(0.63 – 0.72)
	eHLQ21	0.73	(0.67 – 0.78)	0.64	(0.59 – 0.69)
	eHLQ26	0.60	(0.53 – 0.67)	0.76	(0.71 – 0.80)
3. Ability to actively engage with digital services	eHLQ4	0.76	(0.71 – 0.80)	0.78	(0.75 – 0.81)
	eHLQ6	0.83	(0.79 – 0.87)	0.78	(0.74 – 0.81)
	eHLQ8	0.77	(0.76 – 0.81)	0.76	(0.73 – 0.80)
	eHLQ17	0.79	(0.76 – 0.83)	0.75	(0.72 – 0.78)
	eHLQ32	0.78	(0.73 – 0.82)	0.86	(0.83 – 0.89)
4. Feel safe and in control	eHLQ1	0.61	(0.55 – 0.67)	0.53	(0.47 – 0.60)
	eHLQ10	0.78	(0.74 – 0.82)	0.75	(0.71 – 0.79)
	eHLQ14	0.58	(0.52 – 0.64)	0.85	(0.82 – 0.88)
	eHLQ22	0.87	(0.84 – 0.91)	0.78	(0.74 – 0.81)
	eHLQ30	0.86	(0.84 – 0.89)	0.86	(0.83 – 0.89)
5. Motivated to engage with digital services	eHLQ2	0.61	(0.54 – 0.68)	0.76	(0.72 – 0.80)
	eHLQ19	0.81	(0.77 – 0.85)	0.87	(0.84 – 0.90)
	eHLQ24	0.89	(0.86 – 0.91)	0.80	(0.77 – 0.83)
	eHLQ27	0.81	(0.78 – 0.85)	0.81	(0.78 – 0.85)
	eHLQ35	0.89	(0.86 – 0.92)	0.87	(0.84 – 0.89)
6. Access to digital services that work	eHLQ3	0.60	(0.54 – 0.67)	0.60	(0.54 – 0.65)
	eHLQ9	0.72	(0.67 – 0.76)	0.76	(0.73 – 0.80)
	eHLQ16	0.77	(0.73 – 0.81)	0.76	(0.73 – 0.80)
	eHLQ23	0.84	(0.81 – 0.87)	0.78	(0.75 – 0.81)
	eHLQ29	0.78	(0.72 – 0.82)	0.75	(0.71 – 0.78)
	eHLQ34	0.75	(0.70 – 0.79)	0.83	(0.80 – 0.85)
7. Digital services that suit individual needs	eHLQ18	0.70	(0.65 – 0.74)	0.82	(0.80 – 0.85)
	eHLQ28	0.89	(0.85 – 0.92)	0.85	(0.83 – 0.88)
	eHLQ31	0.90	(0.87 – 0.92)	0.84	(0.81 – 0.86)
	eHLQ33	0.86	(0.82 – 0.89)	0.86	(0.84 – 0.88)

All loadings are significant at $p < 0.01$

All factor loadings > 0.5
