# Supplementary material for: Translation, Cultural Adaptation, and Validation of the Japanese eHealth Literacy Questionnaire Among Users in a Super-Aged Society: Mixed Methods Study
Source: J Med Internet Res. 2025 Nov 26;27:e68529. doi: 10.2196/68529 (PMC12661597; doi:10.2196/68529)
Supplement: Multimedia Appendix 3 [file jmir-v27-e68529-s003.pdf]

Multimedia Appendix 3: A confirmatory factor analysis of the Japanese version of eHLQ (a 7-factor model).

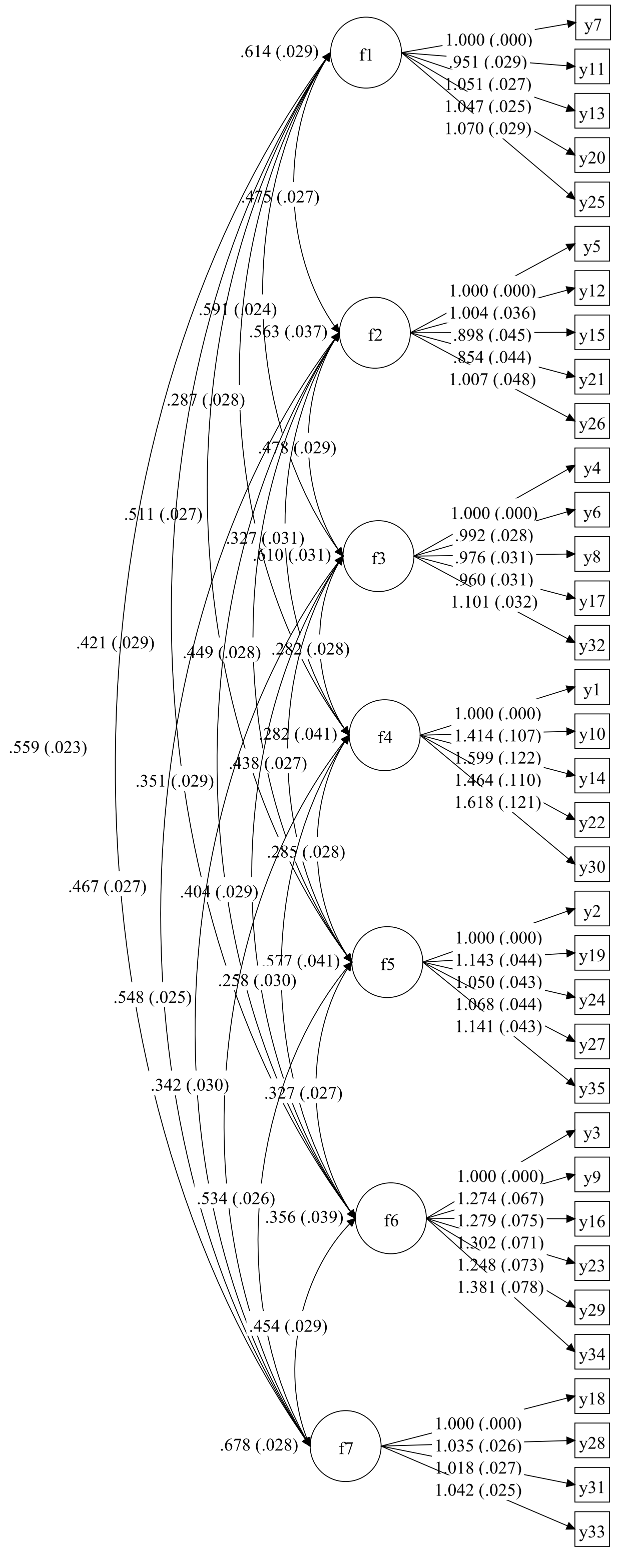

*The first factor indicator is fixed at one
