# Supplementary material for: Translation, Cultural Adaptation, and Validation of the Japanese eHealth Literacy Questionnaire Among Users in a Super-Aged Society: Mixed Methods Study
Source: J Med Internet Res. 2025 Nov 26;27:e68529. doi: 10.2196/68529 (PMC12661597; doi:10.2196/68529)
Supplement: Multimedia Appendix 4 [file jmir-v27-e68529-s004.pdf]

Multimedia Appendix 4. Item response theory (IRT) analysis of the Japanese version of the eHealth Literacy Questionnaire (eHLQ).

Scale	Item	Item location	95% CI	Item discrimination	95% CI
1. Using technology to process health information	eHLQ7	0.09	(-0.039 – 0.23)	2.07	(1.55 – 2.59)
	eHLQ11	0.23	(0.10 – 0.36)	2.38	(1.72 – 3.04)
	eHLQ13	-0.15	(-0.29 – -0.004)	2.01	(1.49 – 2.53)
	eHLQ20	0.24	(0.11 – 0.37)	2.74	(2.08 – 3.39)
	eHLQ25	0.57	(0.40 – 0.74)	2.02	(1.46 – 2.58)
2. Understanding of health concepts and language	eHLQ5	0.10	(-0.06 – 0.25)	1.77	(1.21 – 2.33)
	eHLQ12	0.46	(0.31 – 0.61)	2.19	(1.54 – 2.83)
	eHLQ15	-0.48	(-0.65 – -0.31)	1.59	(1.14 – 2.05)
	eHLQ21	0.03	(-0.14 – 0.19)	1.72	(1.24 – 2.19)
	eHLQ26	0.01	(-0.18 – 0.21)	1.14	(0.74 – 1.55)
3. Ability to actively engage with digital services	eHLQ4	0.27	(0.11 – 0.42)	1.78	(1.28 – 2.28)
	eHLQ6	0.14	(0.02 – 0.27)	2.60	(1.94 – 3.26)
	eHLQ8	0.14	(0.01 – 0.27)	2.07	(1.57 – 2.56)
	eHLQ17	0.26	(0.13 – 0.40)	1.91	(1.38 – 2.44)
	eHLQ32	0.41	(0.26 – 0.57)	1.87	(1.34 – 2.40)
4. Feel safe and in control	eHLQ1	-0.51	(-0.74 – -0.27)	1.21	(0.84 – 1.59)
	eHLQ10	-0.08	(-0.21 – 0.05)	2.31	(1.42 – 3.21)
	eHLQ14	0.84	(0.57 – 1.11)	1.03	(0.74 – 1.31)
	eHLQ22	-0.13	(-0.26 – -0.01)	3.12	(2.21 – 4.03)
	eHLQ30	-0.01	(-0.14 – 0.11)	2.90	(2.19 – 3.61)
5. Motivated to engage with digital services	eHLQ2	-0.13	(-0.34 – 0.068)	1.15	(0.85 – 1.45)
	eHLQ19	-0.26	(-0.39 – -0.13)	2.60	(1.67 – 3.54)
	eHLQ24	-0.42	(-0.55 – -0.29)	3.57	(2.64 – 4.49)
	eHLQ27	-0.16	(-0.29 – -0.03)	2.29	(1.71 – 2.86)
	eHLQ35	-0.42	(-0.54 – -0.29)	3.28	(2.40 – 4.16)
6. Access to digital services that work	eHLQ3	-0.10	(-0.30 – 0.099)	1.15	(0.81 – 1.49)
	eHLQ9	0.25	(0.09 – 0.41)	1.55	(1.15 – 1.94)
	eHLQ16	0.75	(0.59 – 0.90)	1.99	(1.46 – 2.52)
	eHLQ23	0.47	(0.33 – 0.62)	2.87	(2.17 – 3.57)
	eHLQ29	0.76	(0.59 – 0.94)	2.07	(1.49 – 2.65)
	eHLQ34	0.36	(0.21 – 0.51)	1.86	(1.38 – 2.35)
7. Digital services that suit individual needs	eHLQ18	0.51	(0.33 – 0.69)	1.58	(1.07 – 2.09)
	eHLQ28	0.23	(0.10 – 0.35)	3.34	(2.44 – 4.24)
	eHLQ31	0.25	(0.12 – 0.38)	3.72	(2.73 – 4.71)
	eHLQ33	0.12	(-0.01 – 0.25)	2.98	(2.16 – 3.81)
