# Supplementary material for: Translation, Cultural Adaptation, and Validation of the Japanese eHealth Literacy Questionnaire Among Users in a Super-Aged Society: Mixed Methods Study
Source: J Med Internet Res. 2025 Nov 26;27:e68529. doi: 10.2196/68529 (PMC12661597; doi:10.2196/68529)

Multimedia Appendix 5: Boundary characteristic curves of the 35 items of the Japanese version eHLQ.

Scale 1

eHLQ7

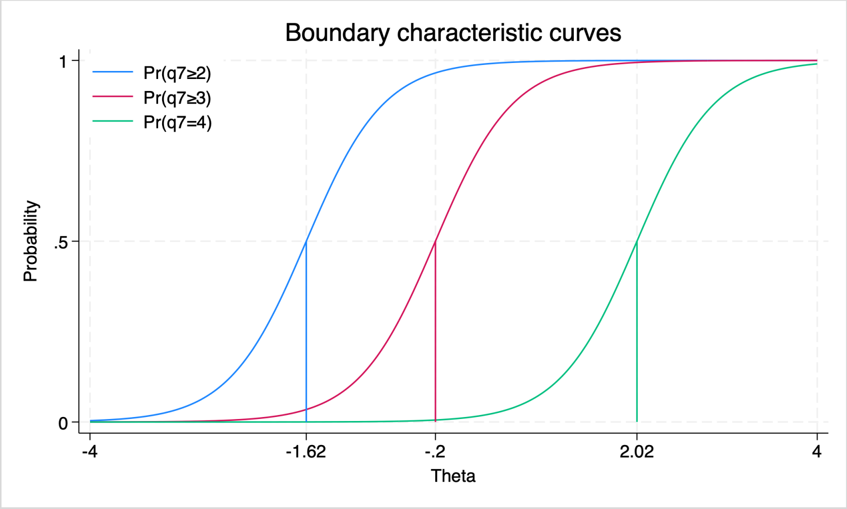

eHLQ11

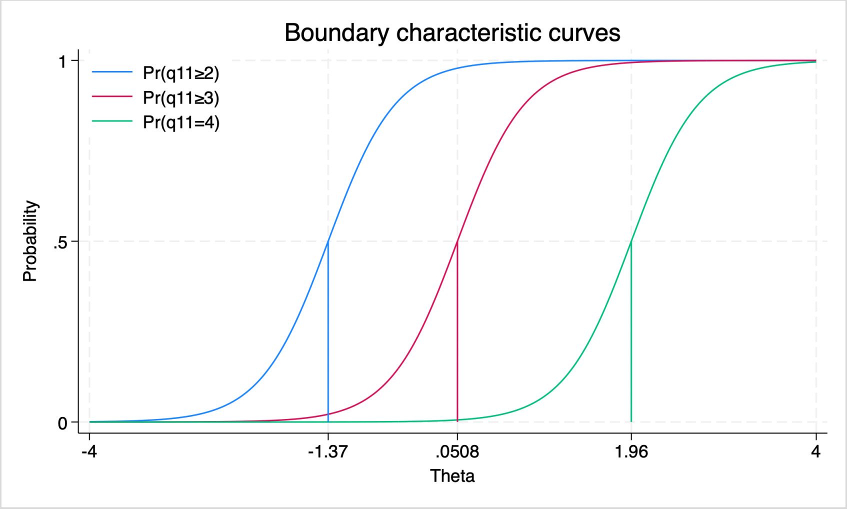

eHLQ13

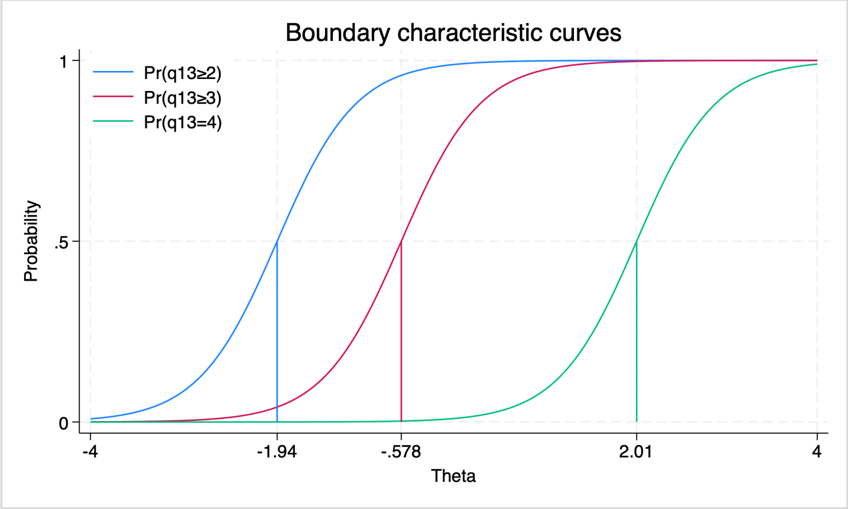

eHLQ20

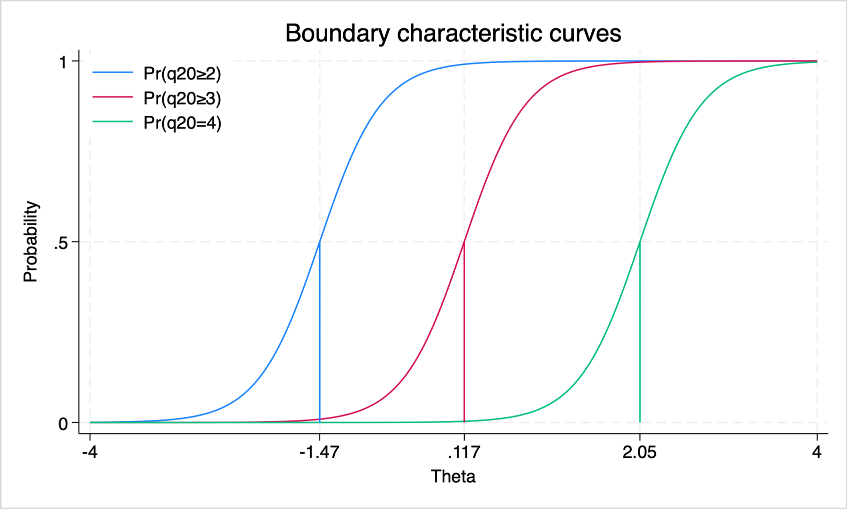

eHLQ25

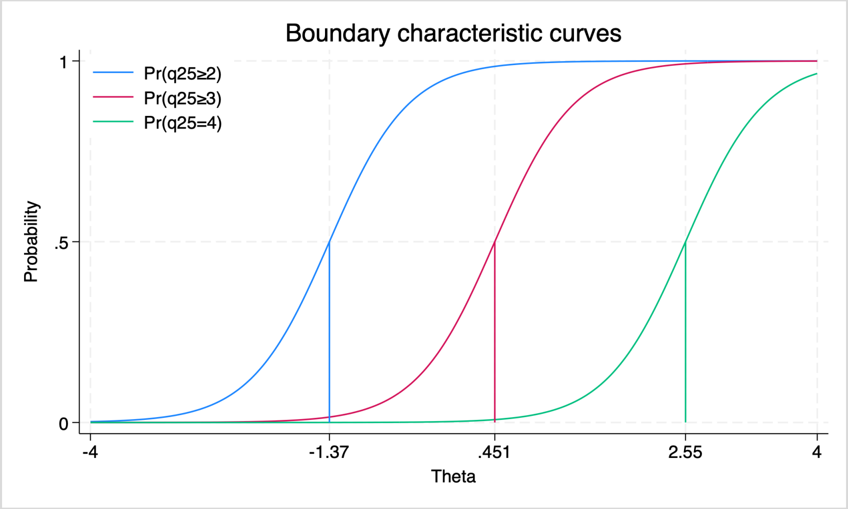

Multimedia Appendix 5

Scale 2

eHLQ5

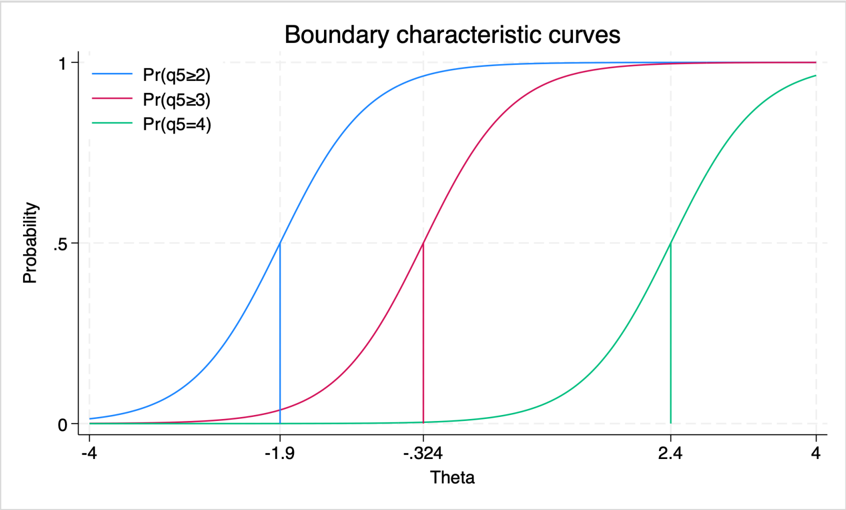

eHLQ12

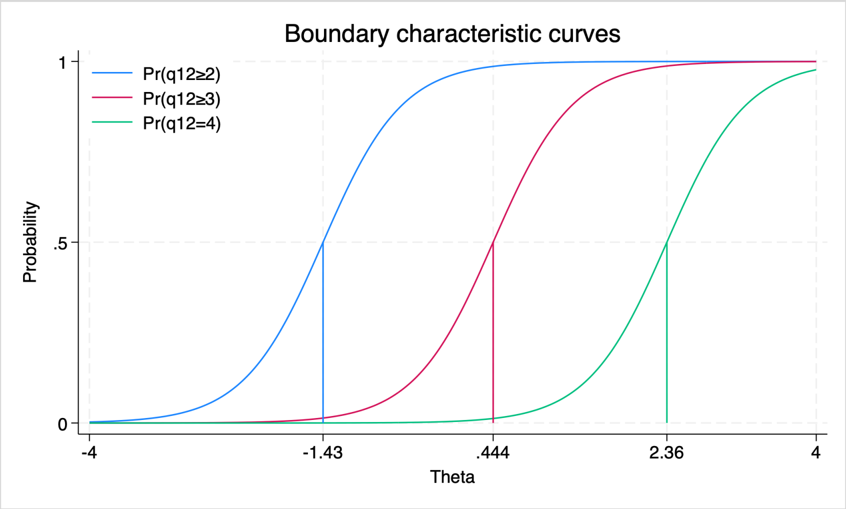

eHLQ15

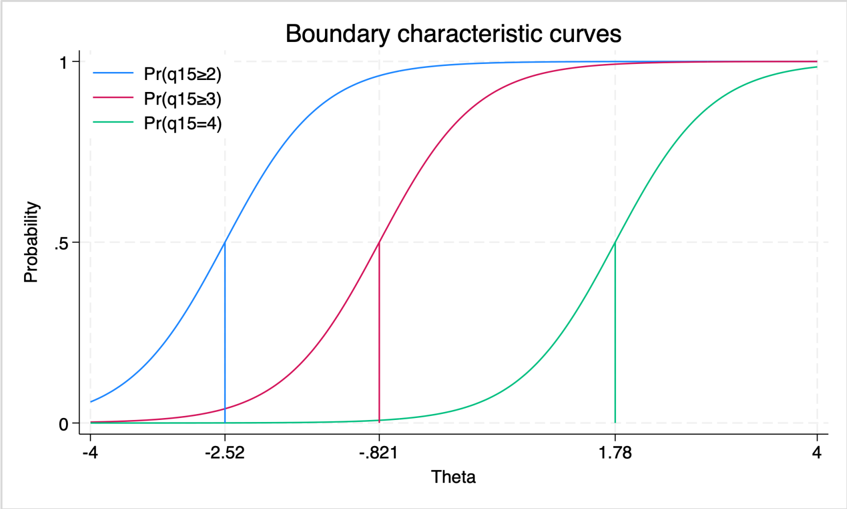

eHLQ21

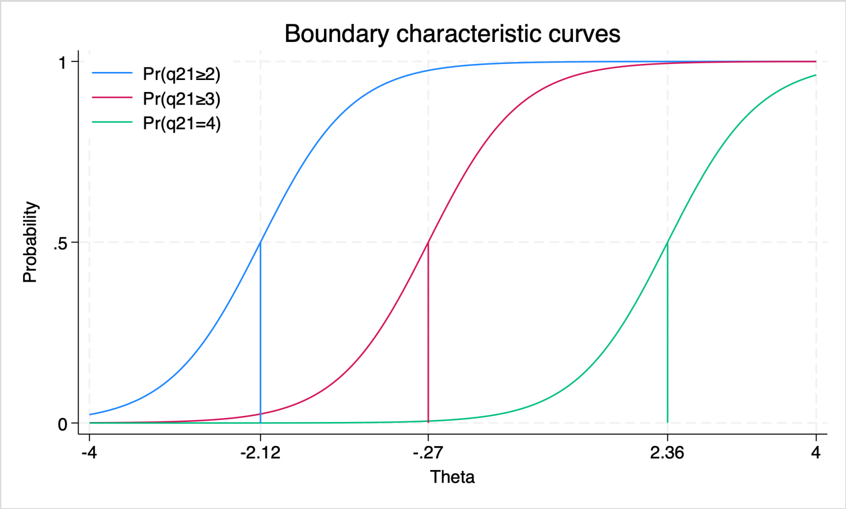

eHLQ26

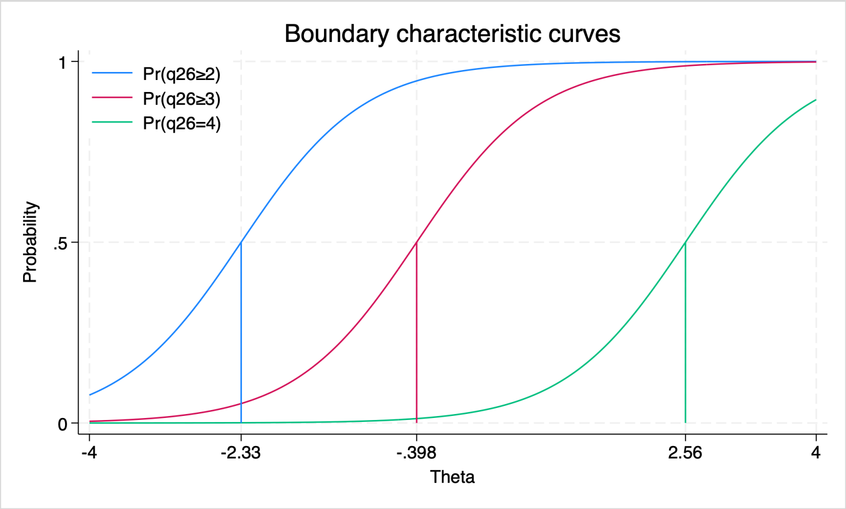

Multimedia Appendix 5

Scale 3

eHLQ4

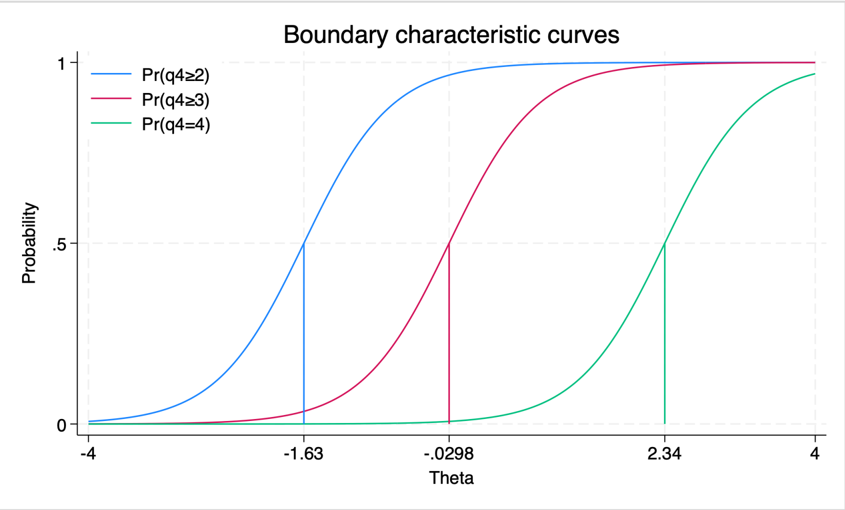

eHLQ6

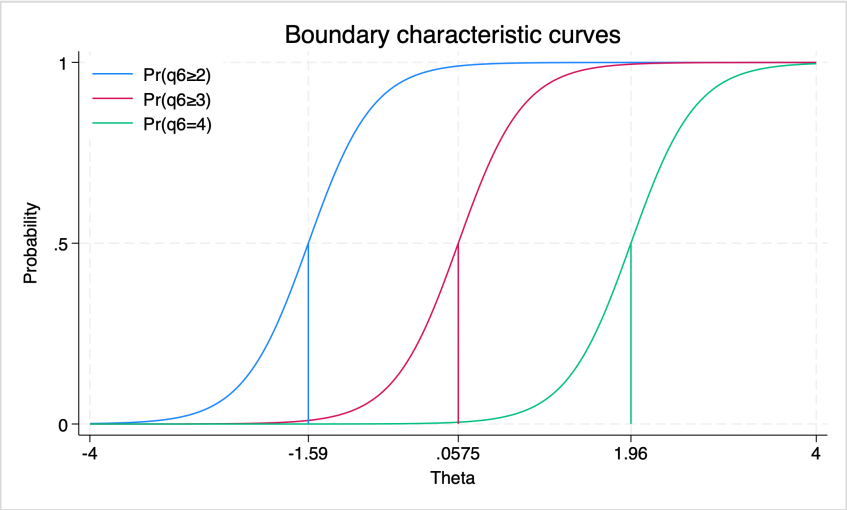

eHLQ8

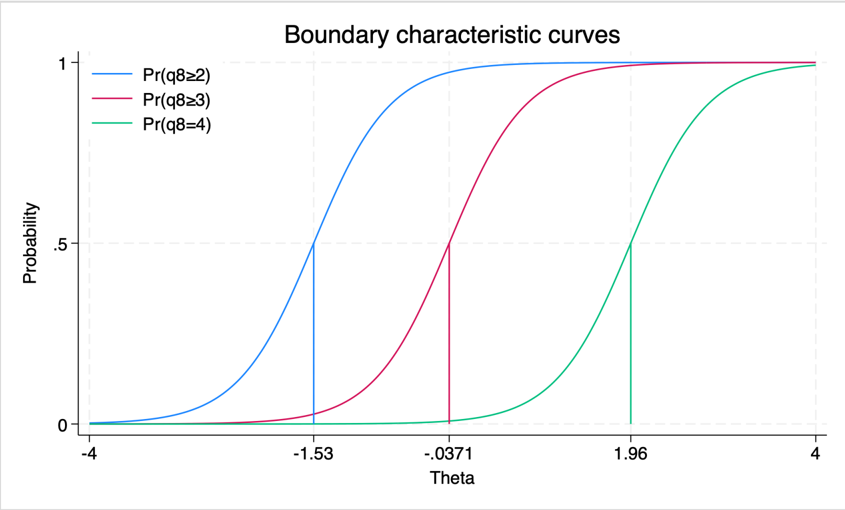

eHLQ17

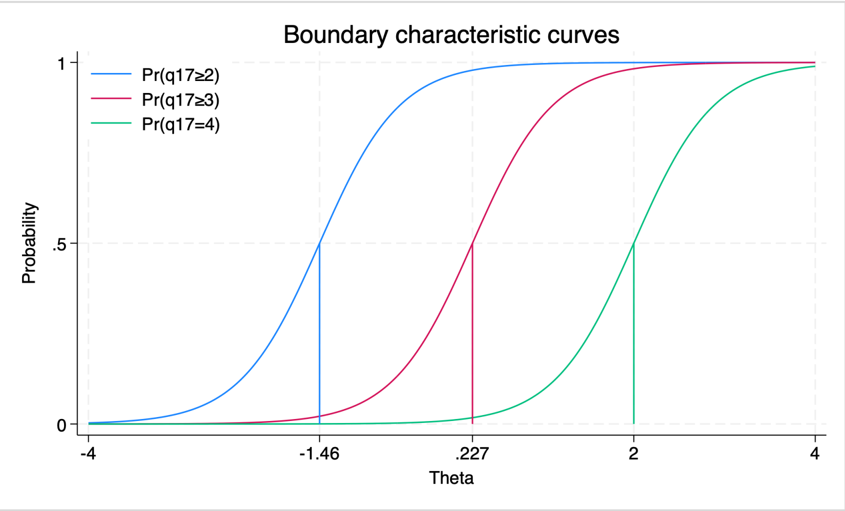

eHLQ32

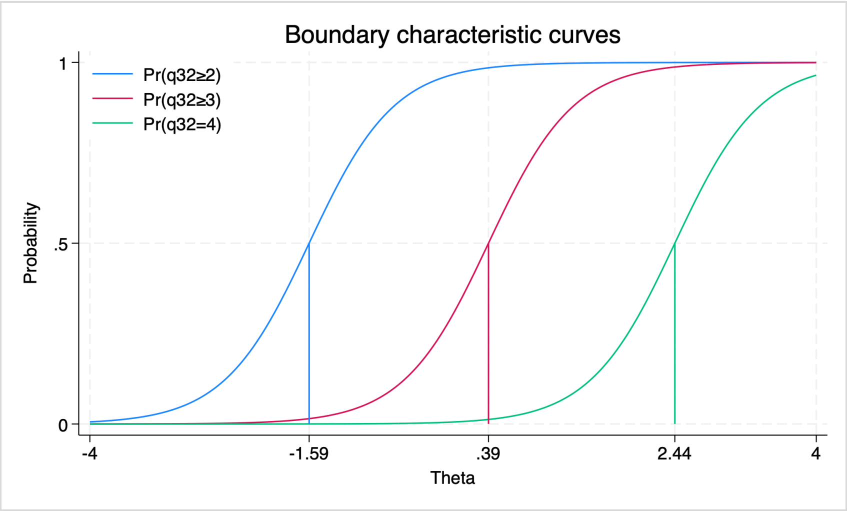

Multimedia Appendix 5

Scale 4

eHLQ1

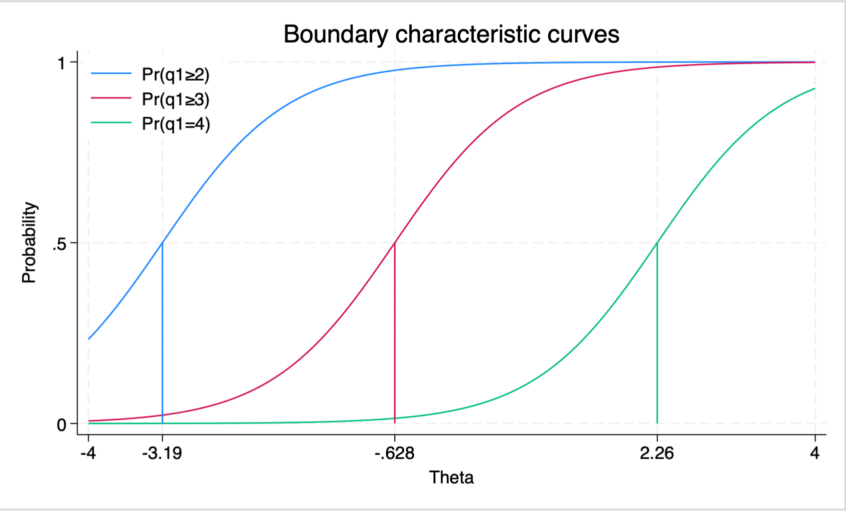

eHLQ10

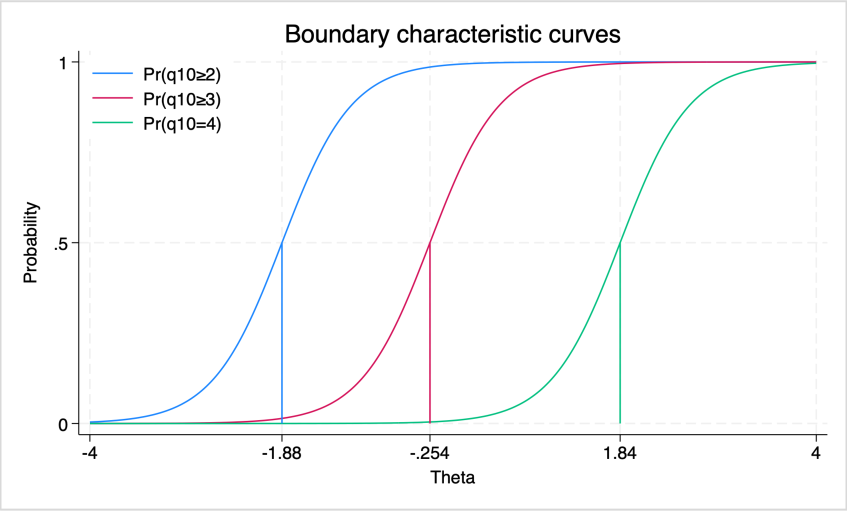

eHLQ14

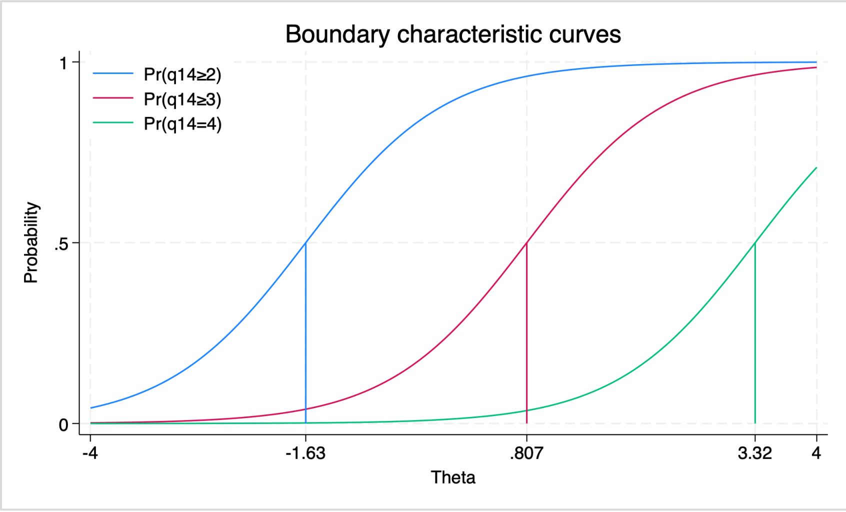

eHLQ22

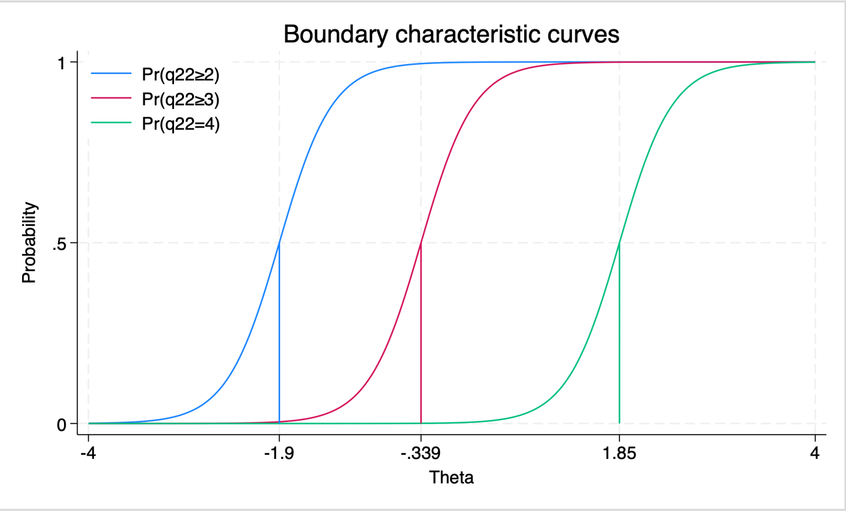

eHLQ30

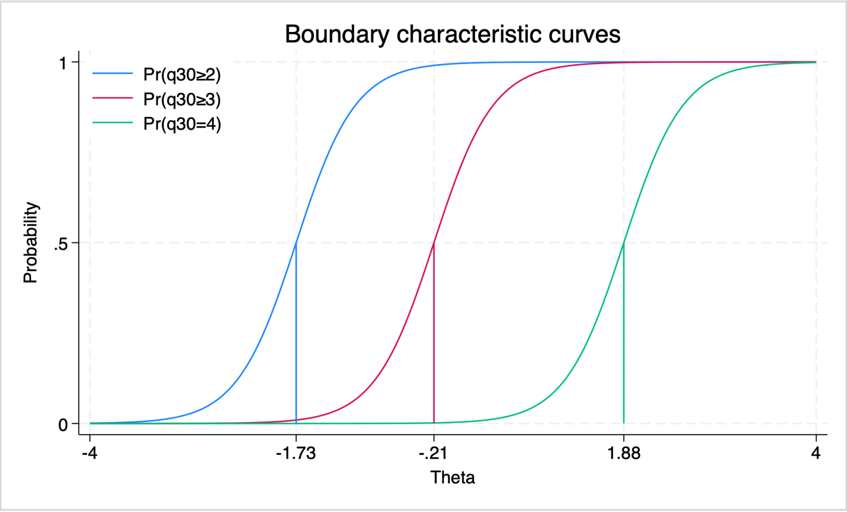

Multimedia Appendix 5

Scale 5

eHLQ2

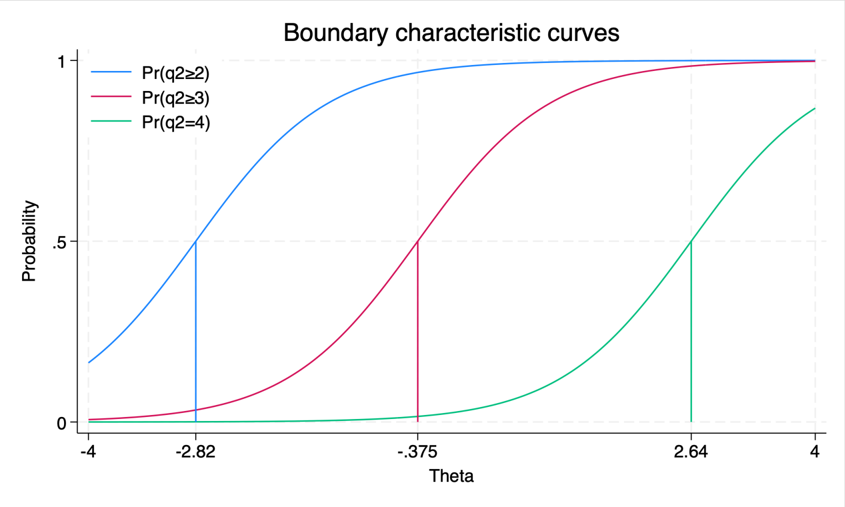

eHLQ19

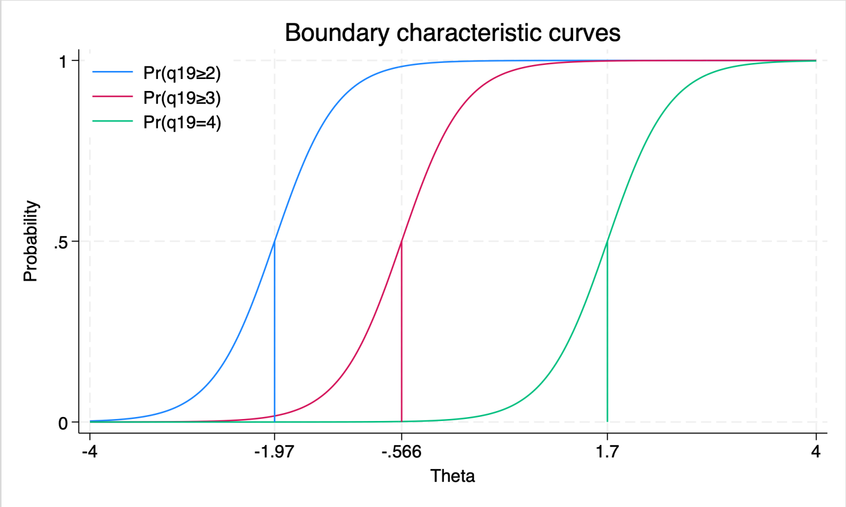

eHLQ24

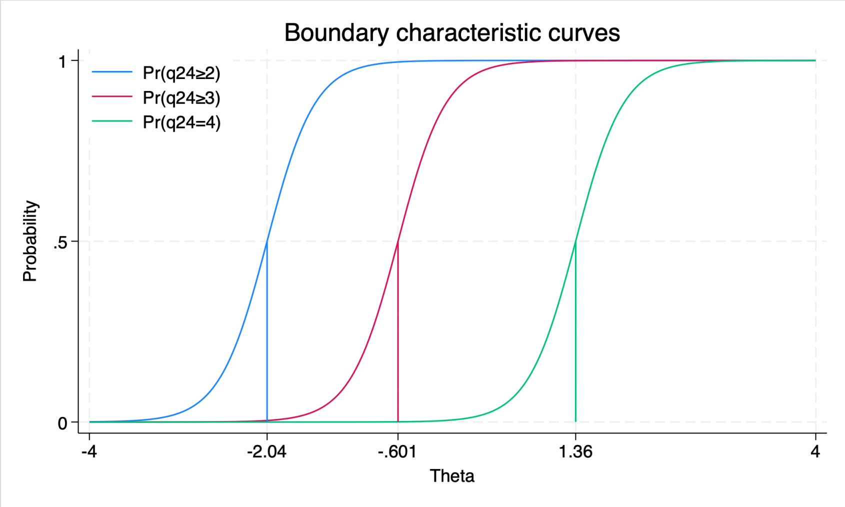

eHLQ27

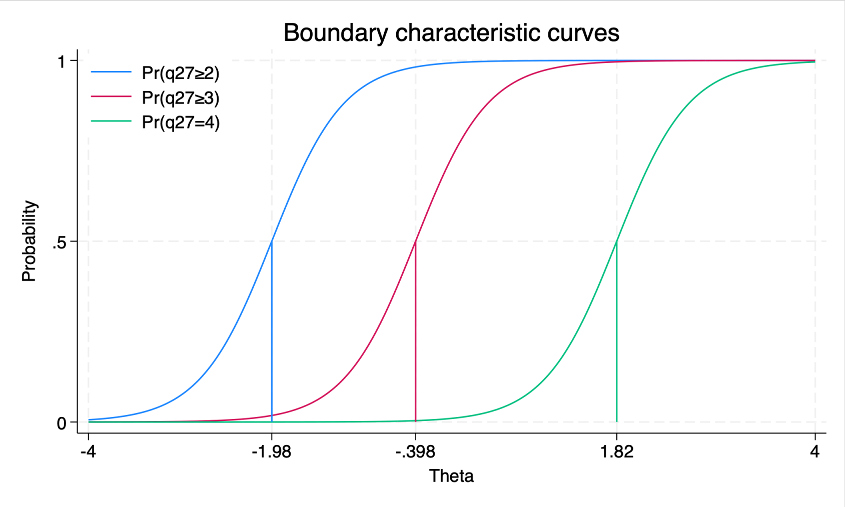

eHLQ35

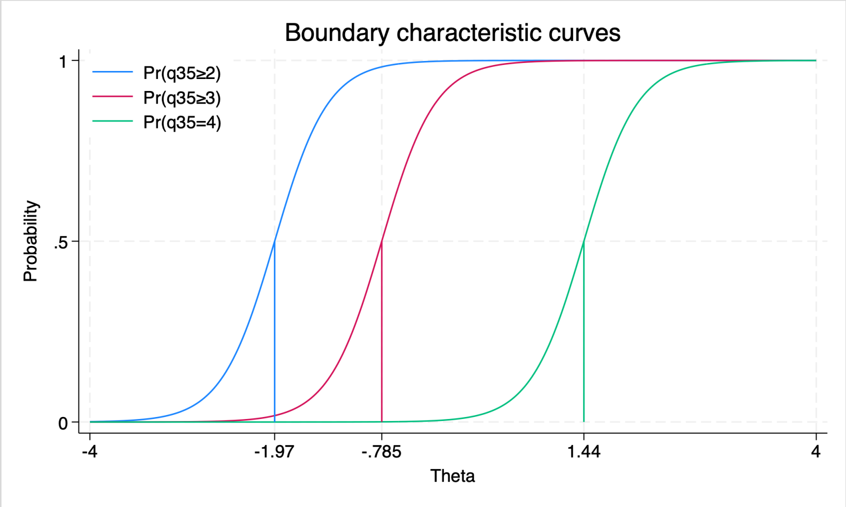

Multimedia Appendix 5

Scale 6

eHLQ3

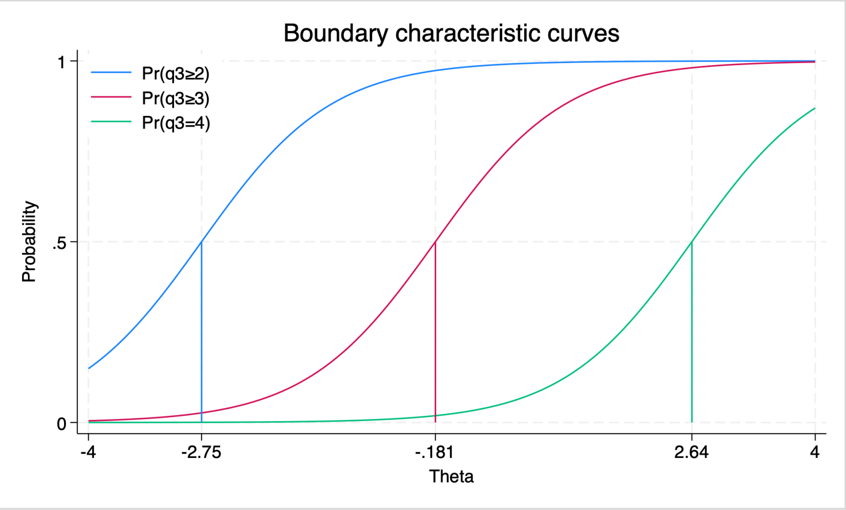

eHLQ9

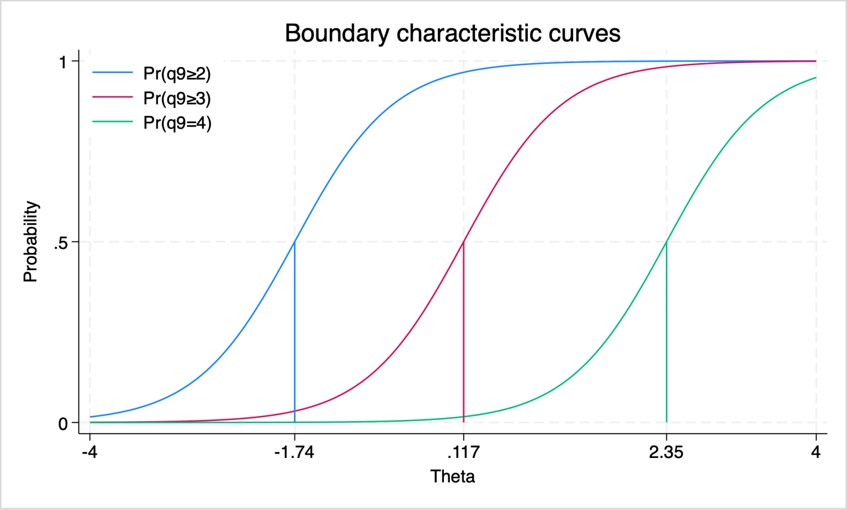

eHLQ16

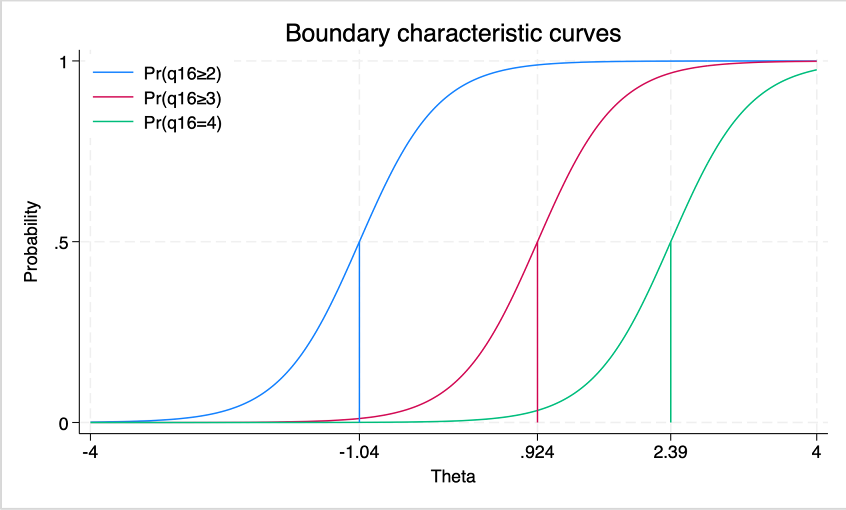

eHLQ23

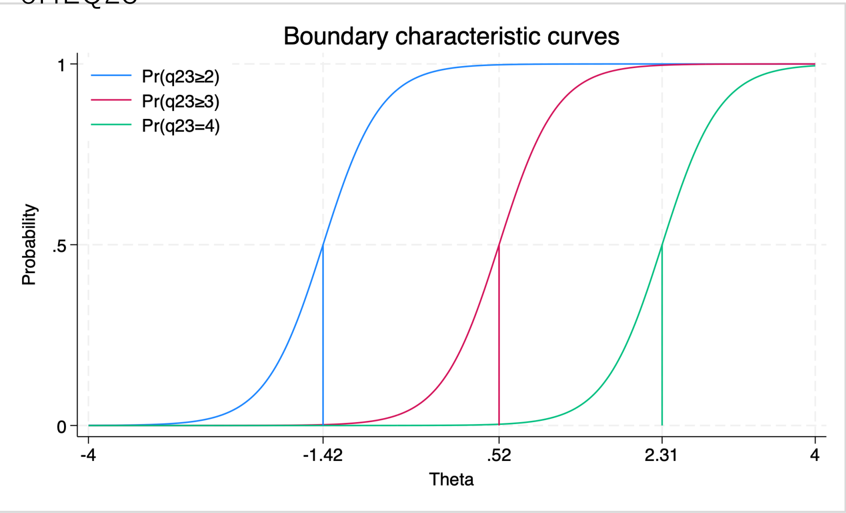

eHLQ29

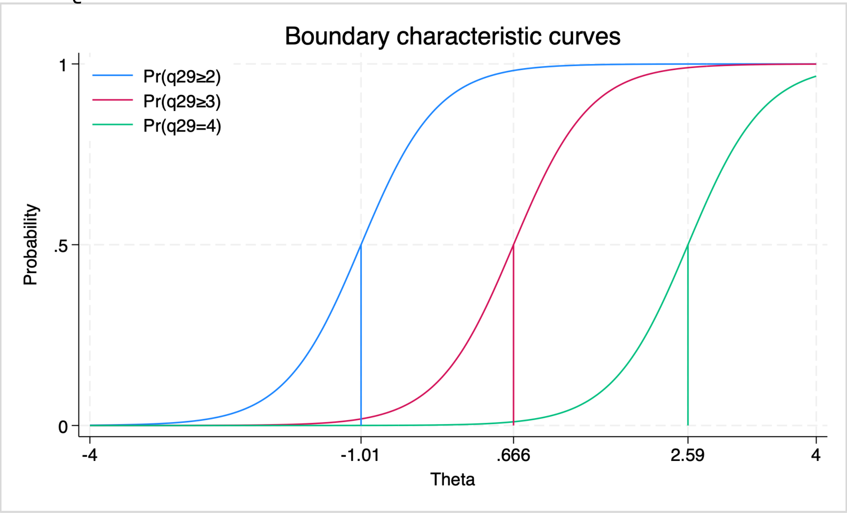

eHLQ34

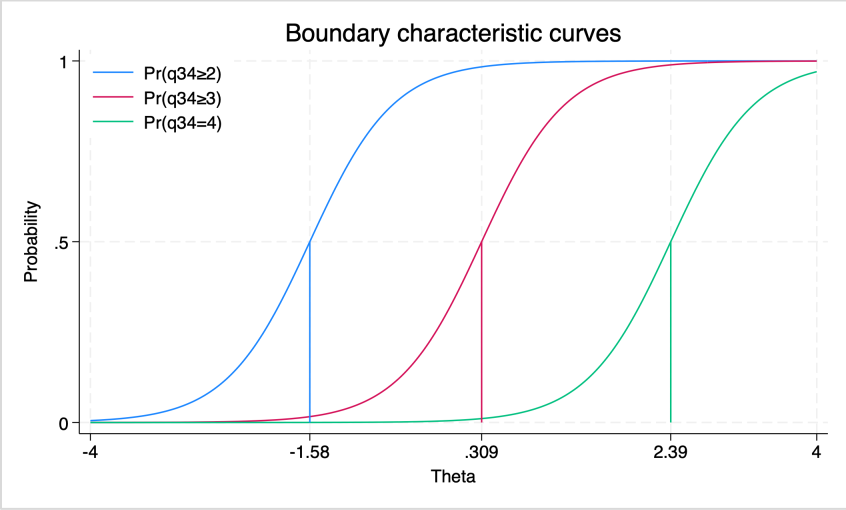

Multimedia Appendix 5

Scale 7

eHLQ18

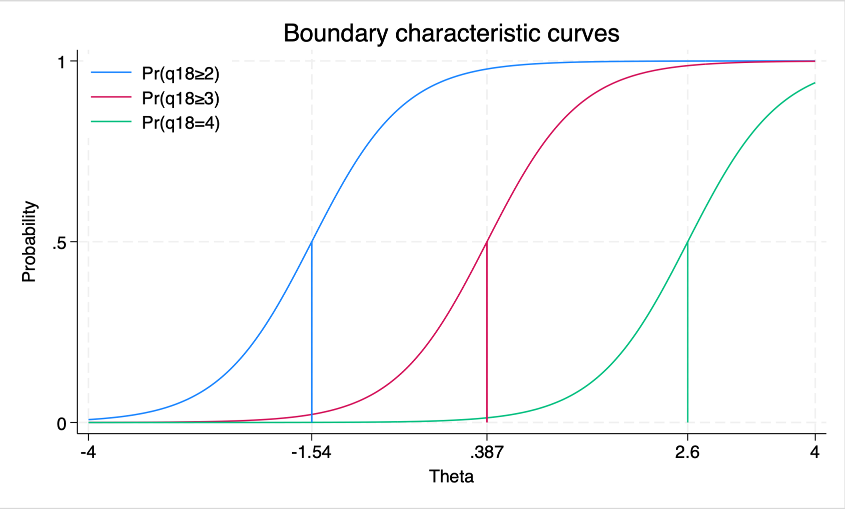

eHLQ28

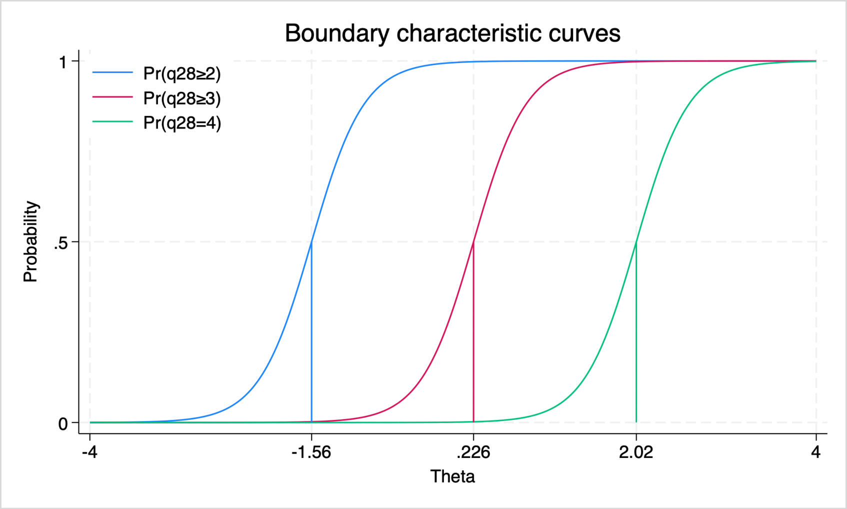

eHLQ31

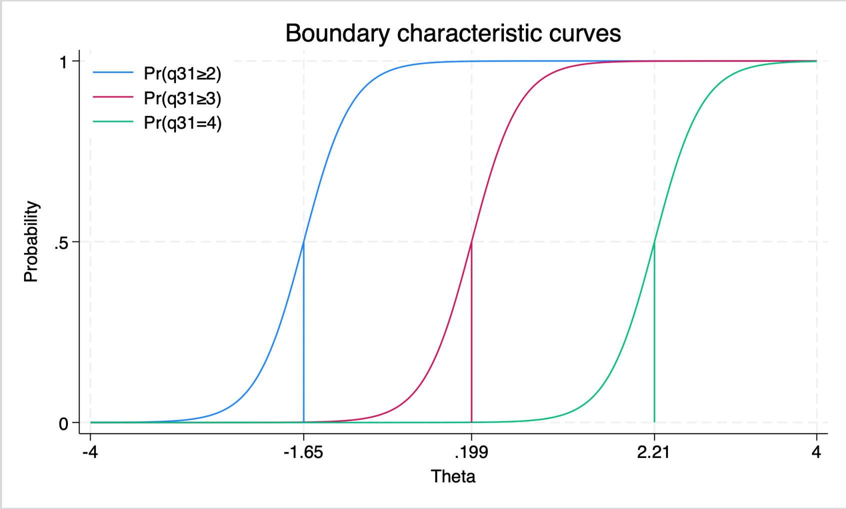

eHLQ33

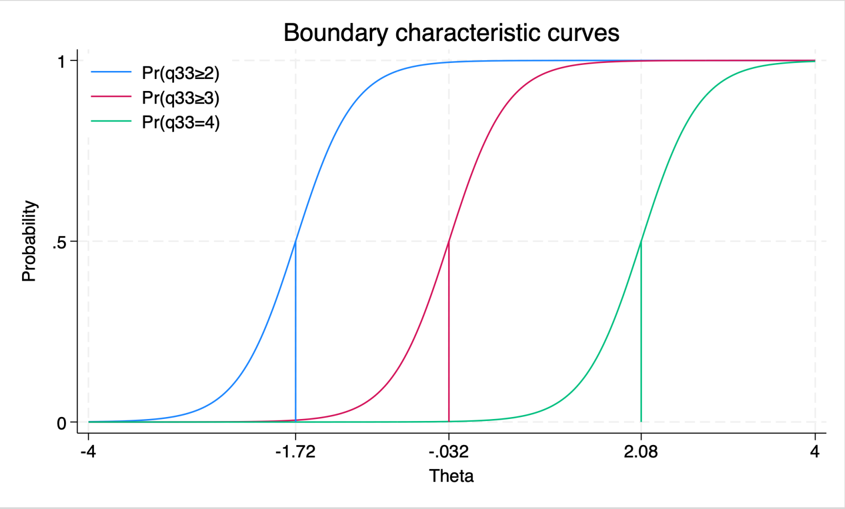

Supplement: Multimedia Appendix 5 [file jmir-v27-e68529-s005.pdf]
