# Supplementary material for: Translation, Cultural Adaptation, and Validation of the Japanese eHealth Literacy Questionnaire Among Users in a Super-Aged Society: Mixed Methods Study
Source: J Med Internet Res. 2025 Nov 26;27:e68529. doi: 10.2196/68529 (PMC12661597; doi:10.2196/68529)
Supplement: Multimedia Appendix 6 [file jmir-v27-e68529-s006.pdf]

Multimedia Appendix 6: Descriptive and comparative statistics for the Japanese version eHLQ scores across demographic groups

1. Residence area

		Variance [n]	mean	std	Post-hoc analysis
**Scale 1**	p-value = **0.030**	1. Hokkaido [22]	2.491	0.481	
		2. Tohoku [46]	2.317	0.523	
Using technology to process health information		3. Kanto [227]	2.544	0.563	
		4. Chubu [53]	2.532	0.623	
		5. Kinki [73]	2.430	0.586	
		6. Chugoku/Shikoku [52]	2.292	0.565	
		7 Kyushu/Okinawa [31]	2.374	0.621	
		Total [504]	2.467	0.575	p-value all > 0.05
**Scale 2**	p-value: 0.864	1. Hokkaido [22]	2.636	0.439	
		2. Tohoku [46]	2.613	0.394	
Understanding of health concepts and language		3. Kanto [227]	2.555	0.491	
		4. Chubu [53]	2.551	0.493	
		5. Kinki [73]	2.548	0.476	
		6. Chugoku/Shikoku [52]	2.538	0.517	
		7 Kyushu/Okinawa [31]	2.458	0.618	
		Total [504]	2.555	0.489	p-value all > 0.05
**Scale 3**	p-value: 0.053	1. Hokkaido [22]	2.482	0.465	
		2. Tohoku [46]	2.248	0.503	
Ability to actively engage with digital services		3. Kanto [227]	2.484	0.581	
		4. Chubu [53]	2.423	0.612	
		5. Kinki [73]	2.351	0.568	
		6. Chugoku/Shikoku [52]	2.277	0.573	
		7 Kyushu/Okinawa [31]	2.290	0.659	
		Total [504]	2.403	0.579	p-value all > 0.05
**Scale 4**	p-value: 0.802	1. Hokkaido [22]	2.436	0.464	
		2. Tohoku [46]	2.635	0.431	
		3. Kanto [227]	2.544	0.518	
Feel safe and in control		4. Chubu [53]	2.570	0.460	
		5. Kinki [73]	2.515	0.525	
		6. Chugoku/Shikoku [52]	2.558	0.538	
		7 Kyushu/Okinawa [31]	2.497	0.651	
		Total [504]	2.545	0.514	p-value all > 0.05
**Scale 5**	p-value: 0.396	1. Hokkaido [22]	2.773	0.406	
		2. Tohoku [46]	2.843	0.389	
		3. Kanto [227]	2.735	0.525	
Motivated to engage with digital services		4. Chubu [53]	2.634	0.524	
		5. Kinki [73]	2.674	0.523	
		6. Chugoku/Shikoku [52]	2.735	0.560	
		7 Kyushu/Okinawa [31]	2.619	0.607	
		Total [504]	2.720	0.519	p-value all > 0.05
**Scale 6**	p-value: 0.465	1. Hokkaido [22]	2.174	0.423	
		2. Tohoku [46]	2.203	0.391	
		3. Kanto [227]	2.343	0.539	
Access to digital services that work		4. Chubu [53]	2.324	0.552	
		5. Kinki [73]	2.322	0.527	
		6. Chugoku/Shikoku [52]	2.259	0.570	
		7 Kyushu/Okinawa [31]	2.210	0.643	
		Total [504]	2.301	0.532	p-value all > 0.05
**Scale 7**	p-value: 0.774	1. Hokkaido [22]	2.318	0.438	
		2. Tohoku [46]	2.451	0.407	
Digital services that suit individual needs		3. Kanto [227]	2.422	0.569	
		4. Chubu [53]	2.448	0.585	
		5. Kinki [73]	2.390	0.592	
		6. Chugoku/Shikoku [52]	2.370	0.563	
		7 Kyushu/Okinawa [31]	2.274	0.663	
		Total [504]	2.404	0.561	p-value all > 0.05

95% CIs: 95% Confidence Interval for Mean

Post-hoc analysis: Bonferroni-adjusted p-values

2-1. Gender (male, female, & other)

		Variance [n]		mean	std	Post-hoc analysis [mean difference; 95% CIs] (effect size)
**Scale 1**	*p-value*: 0.333	1. Male [257]		2.482	0.585	
Using technology to process health information		2. Female [245]		2.456	0.558	
		3. Other [2]		1.900	1.273	
			Total [504]	2.467	0.575	*p*-value all > 0.05
**Scale 2**	*p-value*: 0.385	1. Male [257]		2.576	0.480	
Understanding of health concepts and language		2. Female [245]		2.536	0.486	
		3. Other [2]		2.200	1.697	
			Total [504]	2.555	0.489	*p*-value all > 0.05
**Scale 3**	*p-value*: 0.235	1. Male [257]		2.445	0.577	
Ability to actively engage with digital services		2. Female [245]		2.361	0.572	
		3. Other [2]		2.200	1.697	
			Total [504]	2.403	0.579	*p*-value all > 0.05
**Scale 4**	*p-value*: 0.009	1. Male [257]		2.571	0.530	1vs3: *p* = 0.01 [1.07; 0.20-1.94] (0.34)
Feel safe and in control		2. Female [245]		2.526	0.487	2vs3: *p* = 0.01 [1.03; 0.16-1.90] (0.34)
		3. Other [2]		1.500	0.707	
			Total [504]	2.545	0.514	
**Scale 5**	*p-value*: 0.522	1. Male [257]		2.738	0.524	
Motivated to engage with digital services		2. Female [245]		2.704	0.499	
		3. Other [2]		2.400	1.980	
			Total [504]	2.720	0.519	*p*-value all > 0.05
**Scale 6**	*p-value*: 0.113	1. Male [257]		2.332	0.553	
Access to digital services that work		2. Female [245]		2.273	0.505	
		3. Other [2]		1.667	0.943	
			Total [504]	2.301	0.532	*p*-value all > 0.05
**Scale 7**	*p-value*: 0.109	1. Male [257]		2.425	0.576	
Digital services that suit individual needs		2. Female [245]		2.388	0.539	
		3. Other [2]		1.667	0.943	
			Total [504]	2.301	0.532	*p*-value all > 0.05

95% CIs: 95% Confidence Interval for Mean

Post-hoc analysis: Bonferroni-adjusted *p*-values

Cohen’s d was used to quantify effect sizes

2-2. Gender (male & female)

		Variance [n]		mean	std	Post-hoc analysis
**Scale 1** Using technology to process health information	p-value: 0.62	1. Male [257]		2.482	0.585	
		2. Female [245]		2.456	0.558	
			Total [502]	2.469	0.572	p-value all > 0.05
**Scale 2** Understanding of health concepts and language	p-value: 0.35	1. Male [257]		2.576	0.480	
		2. Female [245]		2.536	0.486	
			Total [502]	2.556	0.483	p-value all > 0.05
**Scale 3** Ability to actively engage with digital services	p-value: 0.10	1. Male [257]		2.445	0.577	
		2. Female [245]		2.361	0.572	
			Total [502]	2.404	0.575	p-value all > 0.05
**Scale 4** Feel safe and in control	p-value: 0.32	1. Male [257]		2.571	0.530	
		2. Female [245]		2.526	0.487	
			Total [502]	2.549	0.509	p-value all > 0.05
**Scale 5** Motivated to engage with digital services	p-value: 0.46	1. Male [257]		2.738	0.524	
		2. Female [245]		2.704	0.499	
			Total [502]	2.721	0.512	p-value all > 0.05
**Scale 6** Access to digital services that work	p-value: 0.22	1. Male [257]		2.332	0.553	
		2. Female [245]		2.273	0.505	
			Total [502]	2.303	0.530	p-value all > 0.05
**Scale 7** Digital services that suit individual needs	p-value: 0.45	1. Male [257]		2.425	0.576	
		2. Female [245]		2.388	0.539	
			Total [502]	2.407	0.558	p-value all > 0.05

95% CIs: 95% Confidence Interval for Mean

Post-hoc analysis: Bonferroni-adjusted p-values

Gender differences showed statistical significance in scale 4; however, this result was influenced by 2 out of 504 people who selected ‘other’ as their gender identity. After excluding these 2 individuals and reanalyzing the data, no statistical significance was found among scores across all seven scales.

3. Degree of urbanization

		Variance [n]		mean	std	Post-hoc analysis [mean difference; 95% CIs] (effect size)
**Scale 1**	p-value: 0.01	1. Special Wards (Tokyo's 23 Wards) [83]		2.586	0.570	1vs3: p = 0.04 [0.20; 0.01-0.39] (0.34)
Using technology to process health information		2. Ordinance-designated city [139]		2.557	0.558	2vs3: p = 0.03 [0.17; 0.01-0.33] (0.05)
		3. City [259]		2.388	0.576	p-value other than above:
		4. Town/Village [23]		2.391	0.554	> 0.05
			Total [504]	2.467	0.575	
**Scale 2**	p-value: 0.41	1. Special Wards (Tokyo's 23 Wards) [83]		2.619	0.501	
Using technology to process health information		2. Ordinance-designated city [139]		2.577	0.496	
		3. City [259]		2.522	0.479	
		4. Town/Village [23]		2.557	0.512	
			Total [504]	2.555	0.489	p-value all > 0.5
**Scale 3**	p-value: < 0.001	1. Special Wards (Tokyo's 23 Wards) [83]		2.576	0.619	1vs3: p < 0.01 [0.27; 0.08-0.46] (0.46)
Using technology to process health information		2. Ordinance-designated city [139]		2.476	0.565	2vs3: p = 0.03 [0.17; 0.01-0.33] (0.17)
		3. City [259]		2.308	0.562	p-value other than above:
		4. Town/Village [23]		2.409	0.541	> 0.05
			Total [504]	2.403	0.579	
**Scale 4**	p-value: 0.46	1. Special Wards (Tokyo's 23 Wards) [83]		2.590	0.529	
Using technology to process health information		2. Ordinance-designated city [139]		2.583	0.540	
		3. City [259]		2.515	0.488	
		4. Town/Village [23]		2.487	0.581	
			Total [504]	2.545	0.514	p-value all > 0.05
**Scale 5**	p-value: 0.30	1. Special Wards (Tokyo's 23 Wards) [83]		2.802	0.540	
Using technology to process health information		2. Ordinance-designated city [139]		2.734	0.508	
		3. City [259]		2.695	0.517	
		4. Town/Village [23]		2.617	0.525	
			Total [504]	2.720	0.519	p-value all > 0.05
**Scale 6**	p-value: 0.04	1. Special Wards (Tokyo's 23 Wards) [83]		2.393	0.508	
Using technology to process health information		2. Ordinance-designated city [139]		2.369	0.533	
		3. City [259]		2.237	0.533	
		4. Town/Village [23]		2.275	0.547	
			Total [504]	2.301	0.532	p-value all > 0.05
**Scale 7**	p-value: 0.32	1. Special Wards (Tokyo's 23 Wards) [83]		2.464	0.582	
Using technology to process health information		2. Ordinance-designated city [139]		2.442	0.564	
		3. City [259]		2.376	0.557	
		4. Town/Village [23]		2.272	0.482	
			Total [504]	2.404	0.561	p-value all > 0.05

95% CIs: 95% Confidence Interval for Mean

Post-hoc analysis: Bonferroni-adjusted p-values

Cohen’s d was used to quantify effect sizes

4. Education level

		Variance [n]	mean	std	Post-hoc analysis [mean difference; 95% CIs] (effect size)
**Scale 1** Using technology to process health information	p-value = 0.04	1. ISCED level ≤3 (High school or below) [163]	2.405	0.571	
		2. ISCED level 5 (Vocational, Junior College) [100]	2.402	0.515	
		3. ISCED level ≥6 (Bachelor's degree or above) [241]	2.536	0.594	
		Total [504]	2.467	0.575	p-value all > 0.5
**Scale 2** Understanding of health concepts and language	p-value < 0.01	1. ISCED level ≤3 (High school or below) [163]	2.483	0.494	3vs1: p = 0.01 [0.15; 0.04-0.27] (0.31)
		2. ISCED level 5 (Vocational, Junior College) [100]	2.472	0.418	3vs2: p = 0.01 [0.17; 0.03-0.30] (0.35)
		3. ISCED level ≥6 (Bachelor's degree or above) [241]	2.637	0.501	1vs2: p = 1.00
		Total [504]	2.555	0.489	
**Scale 3** Ability to actively engage with digital services	p-value < 0.01	1. ISCED level ≤3 (High school or below) [163]	2.317	0.575	1vs3: p = 0.01 [0.18; 0.04-0.32] (0.30)
		2. ISCED level 5 (Vocational, Junior College) [100]	2.326	0.452	2vs3: p = 0.04 [0.17; 0.004-0.33] (0.29)
		3. ISCED level ≥6 (Bachelor's degree or above) [241]	2.494	0.617	1vs2: p = 1.00
		Total [504]	2.403	0.579	
**Scale 4** Feel safe and in control	p-value = 0.28	1. ISCED level ≤3 (High school or below) [163]	2.550	0.485	
		2. ISCED level 5 (Vocational, Junior College) [100]	2.474	0.445	
		3. ISCED level ≥6 (Bachelor's degree or above) [241]	2.571	0.556	
		Total [504]	2.545	0.514	p-value all > 0.05
**Scale 5** Motivated to engage with digital services	p-value = 0.40	1. ISCED level ≤3 (High school or below) [163]	2.753	0.485	
		2. ISCED level 5 (Vocational, Junior College) [100]	2.664	0.472	
		3. ISCED level ≥6 (Bachelor's degree or above) [241]	2.720	0.558	
		Total [504]	2.720	0.519	p-value all > 0.05
**Scale 6** Access to digital services that work	p-value = 0.04	1. ISCED level ≤3 (High school or below) [163]	2.243	0.502	
		2. ISCED level 5 (Vocational, Junior College) [100]	2.243	0.465	
		3. ISCED level ≥6 (Bachelor's degree or above) [241]	2.364	0.572	
		Total [504]	2.301	0.532	p-value all > 0.05
**Scale 7** Digital services that suit individual needs	p-value = 0.21	1. ISCED level ≤3 (High school or below) [163]	2.396	0.519	
		2. ISCED level 5 (Vocational, Junior College) [100]	2.325	0.499	
		3. ISCED level ≥6 (Bachelor's degree or above) [241]	2.442	0.608	
		Total [504]	2.404	0.561	p-value all > 0.05

95% CIs: 95% Confidence Interval for Mean

Post-hoc analysis: Bonferroni-adjusted p-values

Cohen’s d was used to quantify effect sizes

5. Working hours

		Variance [n]	mean	std	Post-hoc analysis [mean difference; 95% CIs] (effect size)
**Scale 1**	p-value = 0.04	1. Unemployed/Retired [151]	2.385	0.564	
Using technology to process health information		2. Less than 20 hours/week [67]	2.421	0.525	
		3. 20-39 hours per week [52]	2.415	0.538	
		4. Full-time (40+ hours/week) [189]	2.557	0.589	
		5. Working hours vary widely from week to week [20]	2.380	0.601	
		6. Other than above (e.g., student) [22]	2.664	0.567	
		7. Do not answer [3]	2.000	1.000	
		Total [504]	2.467	0.575	p-value all > 0.05
**Scale 2**	p-value = 0.25	1. Unemployed/Retired [151]	2.532	0.490	
Understanding of health concepts and language		2. Less than 20 hours/week [67]	2.579	0.459	
		3. 20-39 hours per week [52]	2.450	0.432	
		4. Full-time (40+ hours/week) [189]	2.580	0.505	
		5. Working hours vary widely from week to week [20]	2.650	0.498	
		6. Other than above (e.g., student) [22]	2.645	0.482	
		7. Do not answer [3]	2.067	0.808	
		Total [504]	2.555	0.489	p-value all > 0.05
**Scale 3**	p-value = 0.02	1. Unemployed/Retired [151]	2.301	0.544	4vs1: p = 0.01 [0.22; 0.03-0.41] (0.38)
Ability to actively engage with digital services		2. Less than 20 hours/week [67]	2.325	0.539	p-value other than above:
		3. 20-39 hours per week [52]	2.358	0.551	> 0.05
		4. Full-time (40+ hours/week) [189]	2.520	0.600	
		5. Working hours vary widely from week to week [20]	2.390	0.610	
		6. Other than above (e.g., student) [22]	2.500	0.623	
		7. Do not answer [3]	2.133	0.987	
		Total [504]	2.403	0.579	
**Scale 4**	p-value = 0.05	1. Unemployed/Retired [151]	2.487	0.495	
Feel safe and in control		2. Less than 20 hours/week [67]	2.651	0.480	
		3. 20-39 hours per week [52]	2.554	0.502	
		4. Full-time (40+ hours/week) [189]	2.525	0.540	
		5. Working hours vary widely from week to week [20]	2.540	0.431	
		6. Other than above (e.g., student) [22]	2.818	0.534	
		7. Do not answer [3]	2.200	0.346	
		Total [504]	2.545	0.514	p-value all > 0.05
**Scale 5**	p-value = 0.01	1. Unemployed/Retired [151]	2.675	0.557	
Motivated to engage with digital services		2. Less than 20 hours/week [67]	2.791	0.401	
		3. 20-39 hours per week [52]	2.746	0.477	
		4. Full-time (40+ hours/week) [189]	2.710	0.519	
		5. Working hours vary widely from week to week [20]	2.780	0.655	
		6. Other than above (e.g., student) [22]	2.873	0.456	
		7. Do not answer [3]	2.000	0.600	
		Total [504]	2.720	0.519	p-value all > 0.05
**Scale 6**	p-value = 0.01	1. Unemployed/Retired [151]	2.225	0.507	6vs3: p = 0.03 [0.38; 0.01-0.75] (0.90)
Access to digital services that work		2. Less than 20 hours/week [67]	2.278	0.437	6vs1: P = 0.03 [0.43; 0.02-0.84] (0.75)
		3. 20-39 hours per week [52]	2.173	0.468	p-value other than above:
		4. Full-time (40+ hours/week) [189]	2.379	0.579	> 0.05
		5. Working hours vary widely from week to week [20]	2.234	0.497	
		6. Other than above (e.g., student) [22]	2.606	0.513	
		7. Do not answer [3]	2.111	0.977	
		Total [504]	2.301	0.532	
**Scale 7**	p-value = 0.01	1. Unemployed/Retired [151]	2.328	0.585	
Digital services that suit individual needs		2. Less than 20 hours/week [67]	2.414	0.412	
		3. 20-39 hours per week [52]	2.341	0.542	
		4. Full-time (40+ hours/week) [189]	2.458	0.589	
		5. Working hours vary widely from week to week [20]	2.463	0.540	
		6. Other than above (e.g., student) [22]	2.580	0.508	
		7. Do not answer [3]	2.000	0.866	
		Total [504]	2.404	0.561	p-value all > 0.05

95% CIs: 95% Confidence Interval for Mean

Post-hoc analysis: Bonferroni-adjusted p-values

Cohen’s d was used to quantify effect sizes

6. Age groups

		Variance [n]	mean	std	Post-hoc analysis [mean difference; 95% CIs] (effect size)
**Scale 1**	p-value = 0.01	1. 10s (18-19 y.o.) [8]	2.850	0.351	2vs6: p = 0.01 [0.33; 0.05-0.61] (0.56)
		2. 20s [72]	2.667	0.623	p-value other than above:
		3. 30s [75]	2.536	0.482	> 0.05
Using technology to process health information		4. 40s [77]	2.431	0.603	
		5. 50s [74]	2.416	0.589	
		6. 60s [96]	2.338	0.564	
		7. 70s [94]	2.432	0.567	
		8. 80s [8]	2.425	0.362	
		Total [504]	2.467	0.575	
**Scale 2**	p-value = 0.07	1. 10s (18-19 y.o.) [8]	2.775	0.446	
		2. 20s [72]	2.572	0.617	
		3. 30s [75]	2.509	0.464	
Understanding of health concepts and language		4. 40s [77]	2.468	0.513	
		5. 50s [74]	2.486	0.474	
		6. 60s [96]	2.550	0.471	
		7. 70s [94]	2.670	0.390	
		8. 80s [8]	2.775	0.392	
		Total [504]	2.555	0.489	p-value all > 0.05
**Scale 3**	p-value = 0.01	1. 10s (18-19 y.o.) [8]	2.925	0.501	2vs6: p = 0.01 [0.29; 0.01-0.57] (0.49)
		2. 20s [72]	2.583	0.677	p-value other than above:
		3. 30s [75]	2.467	0.510	> 0.05
Ability to actively engage with digital services		4. 40s [77]	2.387	0.624	
		5. 50s [74]	2.349	0.557	
		6. 60s [96]	2.292	0.518	
		7. 70s [94]	2.357	0.567	
		8. 80s [8]	2.200	0.370	
		Total [504]	2.403	0.579	
**Scale 4**	p-value < 0.01	1. 10s (18-19 y.o.) [8]	3.100	0.262	1vs3: p = 0.02 [0.64; 0.05-1.23] (1.31)
		2. 20s [72]	2.617	0.622	1vs5: p = 0.01 [0.71; 0.12-1.30] (1.47)
		3. 30s [75]	2.461	0.505	7vs5: p = 0.02 [0.27; 0.02-0.52] (0.58)
		4. 40s [77]	2.532	0.562	p-value other than above:
Feel safe and in control		5. 50s [74]	2.395	0.495	> 0.05
		6. 60s [96]	2.521	0.457	
		7. 70s [94]	2.664	0.420	
		8. 80s [8]	2.525	0.413	
		Total [504]	2.545	0.514	
**Scale 5**	p-value = 0.01	1. 10s (18-19 y.o.) [8]	3.075	0.385	7vs5: p = 0.03 [0.26; 0.01-0.51] (0.52)
		2. 20s [72]	2.811	0.624	p-value other than above:
		3. 30s [75]	2.683	0.515	> 0.05
		4. 40s [77]	2.655	0.474	
Motivated to engage with digital services		5. 50s [74]	2.595	0.525	
		6. 60s [96]	2.660	0.498	
		7. 70s [94]	2.851	0.470	
		8. 80s [8]	2.850	0.256	
		Total [504]	2.720	0.519	
**Scale 6**	p-value < 0.01	1. 10s (18-19 y.o.) [8]	2.729	0.436	2vs6: p < 0.01 [0.32; 0.05-0.59] (0.66)
		2. 20s [72]	2.514	0.667	2vs5: p = 0.01 [0.37; 0.12-0.63] (0.54)
		3. 30s [75]	2.276	0.509	p-value other than above:
		4. 40s [77]	2.320	0.557	> 0.05
Access to digital services that work		5. 50s [74]	2.198	0.502	
		6. 60s [96]	2.141	0.477	
		7. 70s [94]	2.344	0.444	
		8. 80s [8]	2.374	0.232	
		Total [504]	2.301	0.532	
**Scale 7**	p-value = 0.01	1. 10s (18-19 y.o.) [8]	2.719	0.388	2vs6: p = 0.01 [0.31; 0.03-0.58] (0.51)
		2. 20s [72]	2.556	0.661	p-value other than above:
		3. 30s [75]	2.423	0.498	> 0.05
Digital services that suit individual needs		4. 40s [77]	2.390	0.588	
		5. 50s [74]	2.321	0.565	
		6. 60s [96]	2.250	0.543	
		7. 70s [94]	2.481	0.506	
		8. 80s [8]	2.375	0.231	
		Total [504]	2.404	0.561	

95% CIs: 95% Confidence Interval for Mean

Post-hoc analysis: Bonferroni-adjusted p-values

Cohen’s d was used to quantify effect sizes

7. Self-reported health status

		Variance [n]		mean	std	Post-hoc analysis [mean difference; 95% CIs] (effect size)
**Scale 1**	p-value < 0.01	1. Very good / good [177]		2.601	0.548	1vs2: p < 0.01 [0.19; 0.06-0.32] (0.35)
Using technology to process health information		2. Nutral [257]		2.410	0.557	1vs3: p < 0.01 [0.26; 0.07-0.46] (0.46)
		3. Bad/ Very bad [70]		2.337	0.643	2vs3: p = 1.00
			Total [504]	2.467	0.575	
**Scale 2**	p-value < 0.01	1. Very good / good [177]		2.682	0.454	1vs2: p < 0.01 [0.17; 0.06-0.29] (0.37)
Understanding of health concepts and language		2. Nutral [257]		2.508	0.472	1vs3: p < 0.01 [0.28; 0.12-0.44] (0.57)
		3. Bad/ Very bad [70]		2.403	0.560	2vs3: p = 0.31
			Total [504]	2.555	0.489	
**Scale 3**	p-value < 0.01	1. Very good / good [177]		2.548	0.600	1vs2: p = 0.01 [0.20; 0.06-0.33] (0.35)
Ability to actively engage with digital services		2. Nutral [257]		2.352	0.539	1vs3: p < 0.01 [0.32; 0.13-0.52] (0.54)
		3. Bad/ Very bad [70]		2.226	0.598	2vs3: p = 0.30
			Total [504]	2.403	0.579	
**Scale 4**	p-value < 0.01	1. Very good / good [177]		2.689	0.498	1vs2: p < 0.01 [0.20; 0.08-0.32] (0.41)
Feel safe and in control		2. Nutral [257]		2.488	0.489	1vs3: p < 0.01 [0.30; 0.13-0.47] (0.58)
		3. Bad/ Very bad [70]		2.389	0.561	2vs3: p = 0.43
			Total [504]	2.545	0.514	
**Scale 5**	p-value < 0.01	1. Very good / good [177]		2.852	0.510	1vs2: p < 0.01 [0.18; 0.06-0.30] (0.37)
Motivated to engage with digital services		2. Nutral [257]		2.669	0.483	1vs3: p = 0.01 [0.28; 0.11-0.45] (0.52)
		3. Bad/ Very bad [70]		2.571	0.599	2vs3: p = 0.47
			Total [504]	2.720	0.519	
**Scale 6**	p-value < 0.01	1. Very good / good [177]		2.435	0.546	1vs2: p < 0.01 [0.20; 0.07-0.33] (0.40)
Access to digital services that work		2. Nutral [257]		2.233	0.480	1vs3: p = 0.02 [0.23; 0.05-0.40] (0.40)
		3. Bad/ Very bad [70]		2.210	0.617	2vs3: p = 0.95
			Total [504]	2.301	0.532	
**Scale 7**	p-value < 0.01	1. Very good / good [177]		2.549	0.572	1vs2: p < 0.01 [0.20; 0.07-0.33] (0.37)
Digital services that suit individual needs		2. Nutral [257]		2.349	0.523	1vs3: p < 0.01 [0.31; 0.13-0.50] (0.54)
		3. Bad/ Very bad [70]		2.236	0.588	2vs3: p = 0.38
			Total [504]	2.404	0.561	

95% CIs: 95% Confidence Interval for Mean

Post-hoc analysis: Bonferroni-adjusted p-values

Cohen’s d was used to quantify effect sizes
